# Exploring Acrylic Acid as an Oxirane Nucleophile: Direct Access to Poly(β‐Hydroxy Acrylates)

**DOI:** 10.1002/cssc.202500575

**Published:** 2025-06-20

**Authors:** Céline Montanari, Lukas Marcos Celada, Wenhao Zhang, Peter Olsén

**Affiliations:** ^1^ Department of Fibre and Polymer Technology KTH Royal Institute of Technology Teknikringen 56 10044 Stockholm Sweden; ^2^ Laboratory of Organic Electronics Wallenberg Wood Science Center Linköping University 60174 Norrköping Sweden; ^3^ Laboratory of Organic Electronics Linköping University 60174 Norrköping Sweden

**Keywords:** acrylic acids, green chemistries, oxiranes, radical polymerizations, transparents

## Abstract

The synthetic freedom to operate is highly dependent on the final application. In polymer science, scalable reactions, simple purification, and the ideal use of renewable and relevant precursors are relied on. This work explores the ring‐opening of oxiranes with acrylic acid (AA) toward β‐hydroxy acrylates; great care is given to the synthetic aspects of the transformation. In addition to its simplicity, and high yield (isolated yield 68%–87%), the methodology is scalable, atom‐economic, and associated with simple purification. Dependent on the initial oxirane, access to a wide range of polymeric properties with a modulus ranging from 0.3 to 630 MPa, strength from 0.3 to 19 MPa, and elongation‐at‐break from 3% to 170% is demonstrated. All four polymers explored are thermally stable above 250 °C and highly transparent. This work emphasizes the potential of AA as a nucleophile for direct access to monomers for a wide range of polymer applications.

## Graphical abstract

Within, we present a scalable, solvent‐free route to β‐hydroxy acrylate monomers using acrylic acid (AA) as a nucleophile with various oxiranes. Once polymerized, the resulting polymers are optically transparent, thermally stable, and range from brittle to ductile. This straightforward method underscores AA's potential in sustainable, bio‐based polymer design.

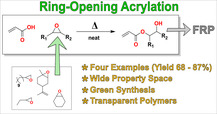

## 1 Introduction

Polymeric materials from renewable resources are central to a future sustainable material economy.^[^ ^1^, ^2^, ^3^, ^4^, ^5^ ^]^ Acrylic monomers are especially important since these building blocks are found in the entire spectrum of polymeric applications, including paints, engineering plastic, composite materials, and biomedical applications. The wide use of acrylics relates to the wide range of properties that depend on the substituent of the repeating units combined with the facile polymerization behavior via either free radical polymerization (FRP), controlled radical polymerization processes, group transfer polymerization, or anionic polymerization. In addition, FRP can even be performed in, or in the presence of, water, making it a highly relevant polymerization method in combination with biopolymers. However, only a tiny portion of the acrylic monomers are of bio‐based origin.^[^ ^6^, ^7^, ^8^ ^]^ Thus, new bio‐based acrylic monomer alternatives are needed to offer a wide range of material properties. To address this, new direct, scalable, monomer synthetic pathways toward acrylic polymers are desirable.

**Figure 1:**
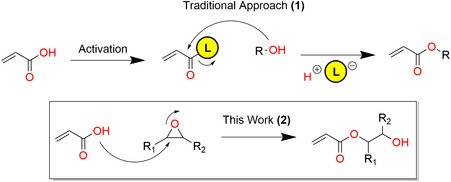
General acrylation strategy explored within this work.

Synthesis of sustainable monomers, in addition to using renewable resources, also necessitates avoiding hazardous chemicals and reducing our solvent consumption.^[^ ^9^, ^10^, ^11^ ^]^ However, as we turn to less reactive reactants, the fundamental challenge is that it becomes harder to control the outcome of the reaction. The lower reactivity requires higher temperatures or a selective catalyst. Ideally, these strategies should also connect to high‐yield, straightforward synthesis and simple purification strategies, particularly in polymer science where scalability is central. Many examples of obtaining renewable methacrylate/acrylate monomers from bio‐based resources exist. Examples include feedstocks such as lignin depolymerization products,^[^ ^12^, ^13^, ^14^ ^]^ terpenes,^[^ ^15^, ^16^ ^]^ and carbohydrates.^[^ ^17^ ^]^ The traditional approach to convert these to monomers is by addition of an activated methacrylation or acrylation electrophile, usually an acid chloride or, activating agent (**Figure** **1**). The essential requirement is that the bio‐based building block, usually an alcohol in combination with a base, is sufficiently nucleophilic to enable the transformation. These synthetic strategies are inherently associated with poor atom economy. Recent advances to address the poor atom economy include using enzymes^[^ ^18^ ^]^ or specific organometallic catalytic strategies.^[^ ^16^ ^]^ It is important to note that to cover a broad property spectrum, direct access and general access to different types of acrylic or methacrylic monomers are required,^[^ ^19^, ^20^ ^]^ and since the final material is in general amorphous, scalability is often more critical than high isomeric purity.

Within, our target is to achieve a monomer synthetic pathway toward acrylates that offer a broad scope of polymeric properties while simultaneously being based on abundant and cheap starting materials, scalable, and easily purified.^[^ ^21^ ^]^ Toward this, we decided to switch the reaction order by, instead of using the acrylic unit as the electrophile, using it as the nucleophile. Our target transformation is the ring‐opening reaction between acrylic acid (AA) and oxiranes (Figure 1). We have previously explored a version of this transformation in our lab toward synthesizing building blocks for fully bio‐based transparent wood and transparent wood for thermal energy storage.^[^ ^22^, ^23^ ^]^ However, general knowledge around the applicability of AA as an oxirane nucleophile is missing. As motivation, bio‐based AA can be obtained from either dehydration of lactic acid, oxidation of acrolein, or even by ethane metathesis on maleic anhydride,^[^ ^24^, ^25^, ^26^, ^27^, ^28^, ^29^ ^]^ even though this pathway still can't compete with petroleum origin in terms of cost. In addition, many bio‐based platform chemicals do not contain alkene functions, these are made readily available through decarboxylation and dehydration.^[^ ^30^, ^31^ ^]^ Alkenes can be oxidized into oxiranes via chloride‐free methods based on either hydrogen peroxide or molecular oxygen.^[^ ^32^, ^33^ ^]^ As such, this work focuses on the ring‐opening acrylation behavior of AA with oxiranes, explores the generality of the chemical transformation, and studies the subsequent polymeric properties in terms of thermal, mechanical, and optical properties.

## 2 Experimental Section

### 2.1 Materials

All the solvents: EtOAc, toluene (≥99%) and dimethylformamide (DMF, ≥99.8%, VWR) were used as received. All the reagents including AA (99%, Sigma Aldrich Sweden), cyclohexene oxide (CO, 98%, Sigma Aldrich), 2,2′‐azobis(2‐methylpropionitrile) (AIBN, 98%, Sigma Aldrich), (+)‐limonene oxide, mixture of cis and trans (LO, 97%. Sigma Aldrich), 2‐(dodecylthiocarbonothioylthio)‐2‐methylpropionic acid (DDMAT, 98%, Sigma Aldrich), 1,2‐epoxydodecane (ED, 90%, Sigma Aldrich), 1,2‐epoxybutane (EB, 99%, Sigma Aldrich), 1‐hydroxycyclohexyl‐phenyl‐ketone (Irgacure 184, 99%, Sigma Aldrich), the catalysts methanesulfonic acid (MSA, 99.5% Sigma Aldrich Sweden), tetrabutylammonium chloride (TBA‐Cl, 97%, Sigma Aldrich), sodium carbonate (Na_2_CO_3_, 99.5%, Sigma Aldrich Sweden), sodium hydroxide (NaOH, 98%, Fisher Scientific), and radical inhibitor 4‐methoxyphenol (99%, Sigma Aldrich) were used as received.

### 2.2 Methods

#### 2.2.1 Ring‐Opening Acrylation of CO with AA Optimization

**Figure 2:**
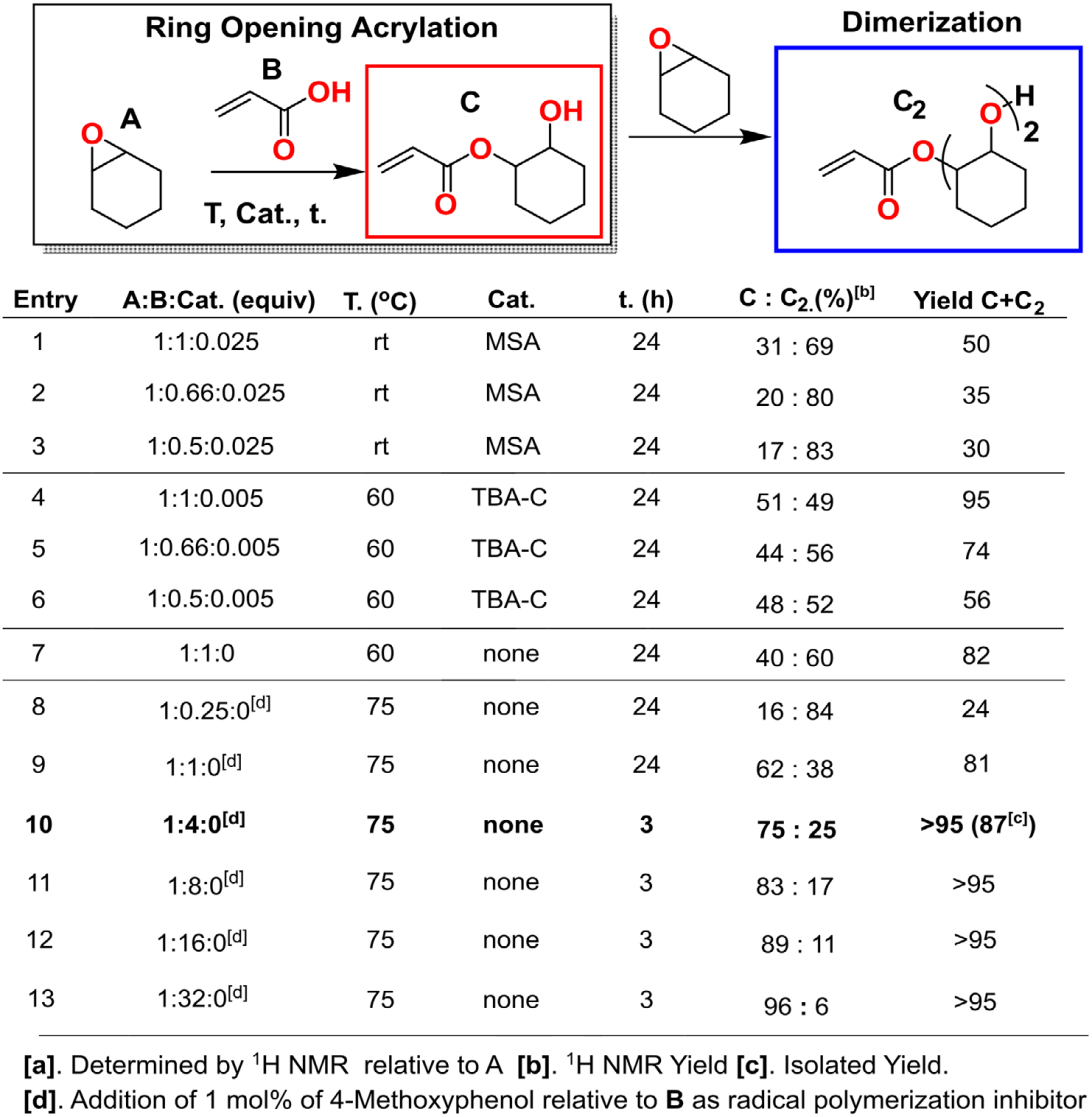
Reaction optimization based on ring‐opening acrylation of cyclohexene oxide with acrylic acid.

In a typical reaction (here entry 9, **Figure** **2**), the desired amount of AA (0.5 g, 6.9 mmol, 1 equiv.) and CO (0.68 g, 6.9 mmol, 1 equiv.) was weighed in a 3 mL vial equipped with a magnetic stirrer. The reaction mixture was immersed in an oil bath and heated to the desired temperature (75 °C). After the desired reaction time (24 h), the reaction mixture was cooled down to ambient temperature and analyzed with ^1^H nuclear magnetic resonance (NMR) for determination of yield. It should be noted that dependent on optimization reaction, either catalyst or radical inhibitor was added to the reaction mixture prior to heating.

#### 2.2.2 Optimized Protocol for Ring‐Opening Acrylation of Different Epoxides with AA

Example reaction between CO and AA. The desired amount of CO (13.6 g, 0.14 mol, 1 equiv.) and AA (40 g, 0.55 mol, 4 equiv.) and 4‐methoxyphenol (0.17 g, 1.4 mmol, 0.01 equiv.) was added to a 100 mL round bottom flask equipped with a magnetic stirrer. The round bottom flask was capped heated at 75 °C for 3 h. After cooling down to ambient temperature, a short distillation column was mounted on the round bottom flask. Temperature was slowly increased to 60 °C while the pressure was reduced to 2–3 mbar. These conditions enabled a dropwise distillation of pure AA from the crude mixture. In a typical experiment, a recovery rate around 95% of the theoretical yield of AA is obtained. The final mixture was decanted into 500 mL beaker with 200 mL NaOH 10 wt% and 200 mL of toluene and a magnetic steric. The mixture poured in a separation funnel and the organic phase was isolated and washed two more times with NaOH (10 wt%) and two times with a brine solution. The organic phase was dried with MgSO_4_(s) and concentrated by rotary evaporation (40 °C) to yield the desired product (2‐hydroxycyclohexyl acrylate) as a transparent oil (26 g, 0.12 mol, 84% yield).

#### 2.2.3 UV‐Initiated FRP

Photoinitiator Irgacure 184 (1 mol%) of was added to around 1 g of each monomer and stirred until fully dissolved. Calculations were carried out by determining an average molar mass for all monomers considering dimerization. Reactive mixtures were then placed between two clamped hydrophobized glass slides. Spacers (0.5 mm thick) were placed in between to ensure uniform thickness between polymeric films. Photopolymerization was then performed using a Fusion UV curing system (F300, Dymax, electrodeless bulbs type BF9, 6.0 J cm^−2^ measured in the interval 320–390 nm). The polymeric film was obtained after 1 min of irradiation. Full acrylate conversion was confirmed by fourier‐transform infrared spectroscopy (FTIR) with the peak disappearing at 1634 cm^−1^ associated with acrylate vinyl group.

#### 2.2.4 Thermal‐Initiated Reversible Addition‐Fragmentation Chain Transfer Polymerization

Initiator AIBN (0.15 mol%) and transfer agent DDMAT (1 mol%) were added to around 0.5 g of purified monomer [2C] in a vial. Toluene (1.5 mL) was injected as solvent. DMF (50 μl) was also added as an internal reference for the determination of conversion by NMR. The mixture was stirred and bubbled with N_2_ gas for 30 min. Then, the reversible addition−fragmentation chain‐transfer (RAFT) polymerization was performed by placing the vial in a heating block at 70 °C. Samples were taken at different reaction time for the kinetic study. The obtained crude products were analyzed by ^1^H NMR and size exclusion chromatography (SEC) in CHCl_3_.

## 3 Results and Discussion

### 3.1 Optimization

The nucleophilic ring‐opening acrylation of oxiranes with AA (B) was optimized with CO (A) as the electrophile. There are exemplified biobased pathways toward 1,4‐cyclohexadiene via self‐metathesis from either cardanol^[^ ^34^ ^]^ or polyunsaturated vegetable oils.^[^ ^35^ ^]^ But, to achieve the targeted oxirane A, 1,4‐cyclohexadiene needs to be selectively mono‐oxidized to cyclohexadiene oxide, followed by reduction of the remaining alkene to oxirane A.^[^ ^36^ ^]^ In addition, chemoenzymatic pathways to reach the cyclohexene directly from oleic acid have been exemplified.^[^ ^37^ ^]^ However, none of these pathways are economically competitive compared to petroleum resources to reach oxirane A. As a oxirane substrate, A is a highly reactive and been used as a homopolymer,^[^ ^38^, ^39^ ^]^ comonomer with either CO_2_,^[^ ^40^, ^41^ ^]^ or cyclic anhydrides.^[^ ^42^, ^43^ ^]^ A was selected based on it high reactivity, and symmetry, resulting in only one regioisomer during the ring‐opening reaction with AA. The nucleophile AA is slightly more acidic than acetic acid,^[^ ^44^ ^]^ with a pKa of 4.1 compared to a pKa of 4.7. Considering this, we foresee similar nucleophilicity between the substrates. Ring‐opening of A with acetic acid is known to proceed under both acidic and basic conditions^[^ ^45^, ^46^ ^]^; however, we could not find any examples of using AA and A. From a monomer synthetic perspective, it is important to have facile purification protocols, ideally, with few by‐products that can be easily removed from the reaction mixture. In the reaction system between A and B, we observed two major products: ring‐opening acrylation toward the desired product C, and further dimerization to product C_2_ (Figure 2).^[^ ^47^ ^]^

The use of reaction solvent increases the E factor of the reaction, regardless of solvent type. Therefore, the first attempt was to perform the reaction under neat conditions under acid‐catalyzed conditions with MSA (2.5 mol% to A) under ambient conditions, varying the ratio of A to B from 1 to 0.5, see Figure 2, entries 1–3. Under these conditions, we obtained the targeted product but also a significant degree of dimerization C_2_, see Figure 2, entries 1–3, but with a 50%–30% yield. Quaternary ammonium salts, such as tert‐butyl ammonium halides, are known to catalyze the ring‐opening of oxiranes.^[^ ^48^ ^]^ The reaction was performed with TBA‐Cl at 60 °C under neat conditions, see Figure 2, entries 4–6. These conditions increased the conversion and reduced the degree of dimerization compared to MSA‐catalyzed conditions. However, when performing the background reaction, it was found that the uncatalyzed condition gave a similar reaction outcome, see entries 4 and 7 (Figure 2). Since removing the catalyst requires an additional purification step, we proceeded with uncatalyzed conditions at a slightly elevated temperature (75 °C) and varied the equivalents of A and B from 0.25 to 32 to study the effect on the degree of dimerization (entries 8–13).

**Figure 3:**
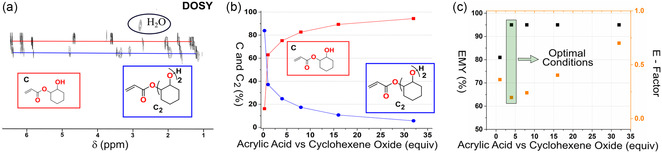
a) DOSY experiment revealing different levels of dimerization and b) product distribution as a function of acrylic acid equiv. against cyclohexene oxide. c) Green chemistry evaluation of the reaction, the effective mass yield (EMY) calculated by atom economy times yield, whereas E‐factor was determined by total mass of waste/mass of product, under the assumption that 90% of unreacted acrylic acid can be recovered.

Increasing the amount of AA (B) relative to oxirane (A) both decreased the degree of dimerization and shortened the reaction time, see Figure 2. During the optimization, excessive B was removed by washing with Na_2_CO_3_ (aq). The dimerized product can be seen by diffusion ordered spectroscopy (DOSY) NMR (**Figure** **3**a). The degree of dimerization was highly dependent on the content of A in relation to B, see Figure 3b and S13, Supporting Information. Figure 3c shows the optimization as a function of effective mass yield (EMY) that is the combination of atom economy and yield, and E factor. The E factor is calculated assuming recovery of 90% of unreacted B. Figure 3c reveals that the reaction conditions most favorable from the perspective of EMY and E factor was using 1 equiv. of A relative to 4 equiv. of B, resulting in an EMY of 95% and an E factor of 0.16. Obviously, this only shows two dimensions of the synthesis and many other factors such as reaction time, purification cost, product purity, etc., are important. However, since here the target is polymeric materials through FRP, we believe the overall EMY and E factor are more critical than the ratio of monomer and dimer. Thus, the optimal condition selected for the substrate scope was 1 equiv. of oxirane relative to 4 equiv. of AA.

### 3.2 Substrate Scope

**Figure 4:**
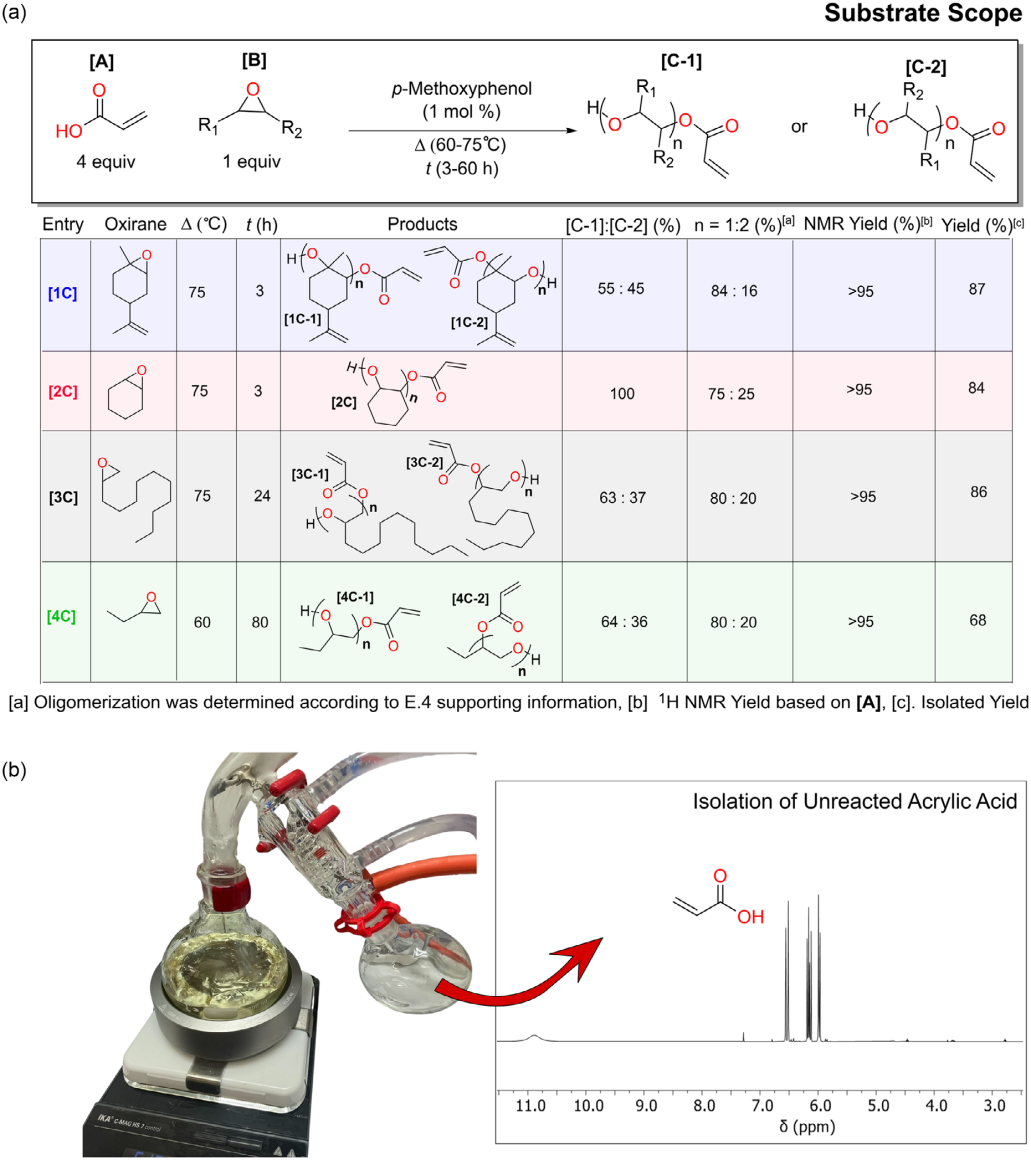
Substrate scope and large‐scale purification. a) Substrate scope of ring‐opening acrylation with a series of renewable epoxides and b) isolation of unreacted acrylic acid via a distillation.

To explore the generality of the protocol, we selected four different oxiranes (LO, cyclohexane oxide, ED [possible biopathways from dodecanol], and EB [possible biopathways from butene]), that would translate to completely different polymeric properties. Although all the oxiranes might be obtained via biobased pathways, the only commercial biobased oxirane is LO. To our delight, using AA as a nucleophile toward oxiranes was found to be general and enabled the direct synthesis of four β‐hydroxy acrylate monomers with moderate to high yields, between 68% to 87%, at a 20–40 g scale. The accessibility of the oxirane ring influenced the rate of reaction (compare entries [1C] and [2C] with [3C] and [4C] in **Figure** **4**a). Therefore, to get high conversion for substrate [3C], the reaction time needed to be increased from 3 to 24 h. For substrate [4C], the reaction temperature was lowered to 60 °C, for 80 h, due to the low boiling point of the oxirane. The correlation between oxirane accessibility and reaction rate is also observed in the literature for the copolymerization of anhydrides and epoxides.^[^ ^49^ ^]^ The amount of dimerized oxirane was similar for all substrates, ranging from 16% to 25%; for insights into the analysis, Figure S1–S28, Supporting Information. All acrylates were synthesized on a 20–40 g scale, and unreacted AA was isolated via distillation at above 90% of the theoretical yield, see Figure 4b. Important to note, it is possible to further reduce the degree of oligomerization by using a higher loading of AA to oxirane; however, this also lowers the overall E factor of the synthesis (Figure 3c).

### 3.3 Polymerization Behavior and Thermal Properties

**Figure 5:**
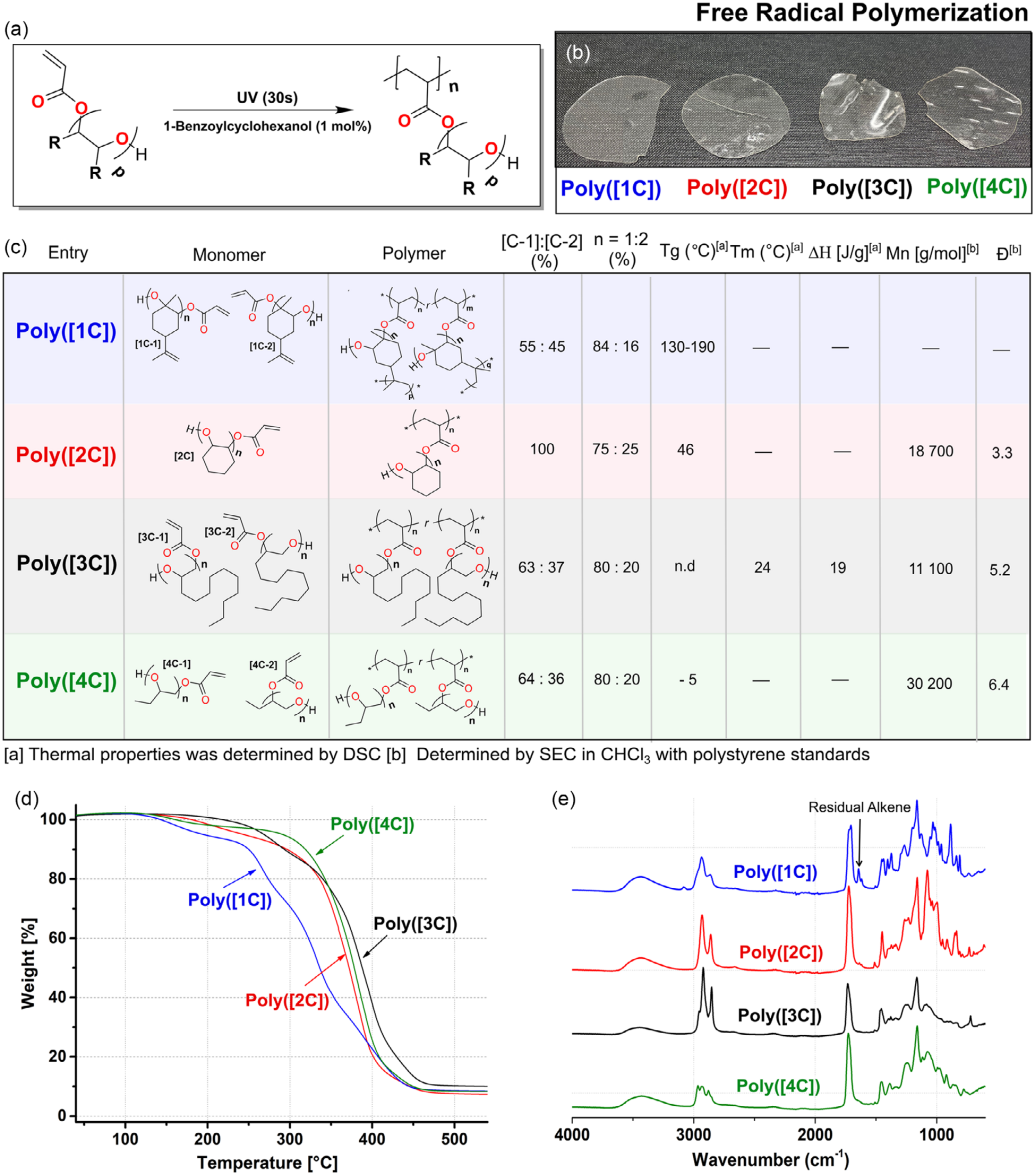
Polymerization and thermal properties. a) Polymerization setup for UV‐initiated free‐radical polymerization, b) visual features of the polymers, and c) physical and thermal properties of the polymers, where the glass transition temperature (Tg) and melting temperature (T_m_) were obtained from differential scanning calorimetry. d) Thermal gravimetrical analysis of the polymers and e) FTIR spectra of the polymers.

The four β‐hydroxy acrylates were polymerized by FRP under UV‐initiated conditions, see **Figure** **5**a, resulting in four poly(β‐hydroxy acrylate) polymers, namely poly(limonene β‐hydroxy‐acrylate) (poly([1C])), poly(cyclohexane β‐hydroxy‐acrylate) (poly([2C])), poly(dodecane β‐hydroxy acrylate) (poly([3C])), and poly(butane β‐hydroxy‐acrylate) (poly([4C])). UV‐initiated polymerization was selected due to its energy efficiency, low‐temperature initiation, precision, and control. Still, for all polymers, we observed partial cross‐linking and high dispersity of the polymers, for more details, see Figure 5 and S29–S31, Supporting Information. Our interpretation is that the high reactivity of the acrylates, in combination and accessible secondary hydroxyl functions in the polymeric chain, gives rise to a higher degree of radical transfer reactions and combinations. Similar observations have been made for the FRP of AA in isopropanol.^[^ ^50^ ^]^ In addition, small degree of diacrylation, although a minor product, will also influence the degree of cross‐linking. FTIR analysis of the final polymers, Figure 5e, shows that the peak associated with alkene stretch at around 1640 cm^−1^ disappeared for all polymers, indicative of high monomer conversion, except for poly([1C]) that has remaining alkene. The remaining alkene stretch in poly([1C]) relates to the two different alkene functions of the monomers, one electron poor relating to the acrylic function and one electron rich relating to the inherent structural features of limonene. During FRP, these units propagate very differently, where the electron‐poor alkene is consumed first, followed by the electron‐rich alkene, resulting in a cross‐linked polymeric material.

The variation in the repeating units resulted in polymeric materials of diverse thermal properties (Figure 5c). The polymers poly([1C]) and poly([2C]) demonstrate a brittle behavior with a Tg above ambient temperature. The Tg for poly([1C]) was in the range of 130 to 190 °C and related to differences in cross‐linking degree from the electron‐rich alkene between the chains, whereas poly([2C]) has a Tg of 46 °C. Poly([3C]) was found to be semicrystalline, with a Tg of −29 °C. The semicrystallinity is attributed to the long aliphatic side‐chain crystalizing, as previously described for long aliphatic acrylates and methacrylates.^[^ ^51^ ^]^ Poly([4C]) was ductile with a low Tg of −5 °C. Comparably, the Tg of the polymer presented falls between polyoxiranes and polyacrylates previously reported for polymerization of the corresponding oxirane or the acrylate mono‐hydroxy version. The higher Tg here compared to polyacrylate polymers is believed to originate from the secondary interaction relating to the β‐hydroxy function. In all cases, the thermal gravimetrical analysis (TGA) shows an on‐set degradation above 250 °C for all these polymeric materials except poly([1C]) under an N_2_ atmosphere (Figure 5d).

### 3.4 Mechanical and Optical Properties

**Figure 6:**
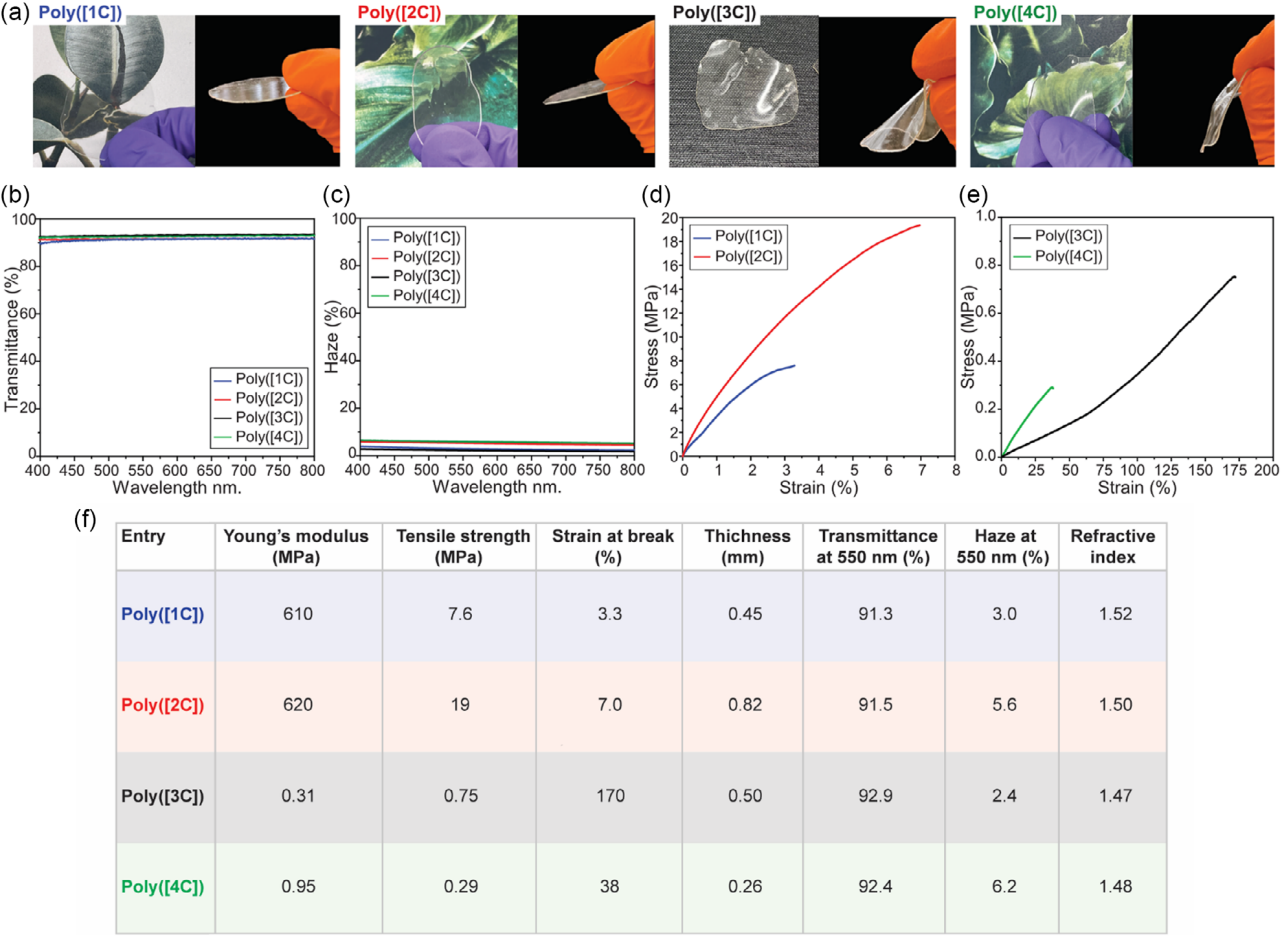
Optical properties and mechanical performance. a) Photographs of the various polymeric films. b) Optical transmittance and c) haze of the polymers. Stress–strain curves of d) poly([1C]) and poly([2C]) and e) poly([3C]) and poly([4C]). f) Table summarizing the mechanical and optical properties of the polymers.

The optical properties were measured by UV–vis with an integrating sphere from 250 to 800 nm. All the polymers are highly transparent, see **Figure** **6**a. The optical transmittance was above 91% for all β‐hydroxyl acrylate polymers (Figure 6b). The haze, corresponding to light‐scattering at wide angles, was very low with values below 7% at 550 nm resulting in a clear optical performance (Figure 6c). It is important to note that no surface treatment was performed to reduce light scattering. The obtained polymers exhibit different refractive index (see Figure 6f), ranging from 1.48 to 1.52.

The mechanical properties of the different Poly(β‐hydroxy acrylates) ranged from stiff and brittle to soft and ductile materials, dictated by the difference in *T*_g_. Both poly([1C]) and poly([2C]) demonstrate a stiff and brittle behavior (Figure 6d). Poly([2C]) shows a tensile strength of 19 MPa, Young's modulus of 620 MPa, and a strain at break of 7%, whereas poly([1C]) had a similar modulus of 610 MPa, but lower strength of 7.6 MPa and more brittle with a strain at break of 3.3.% (Figure 6d–f). The increased brittleness poly([1C]) compared to poly([2C]) is believed to arise from the di‐functionality of the monomer that results in a heterogeneous cross‐linked network. In contrast, both poly([3C]) and poly([4C]) are highly ductile (Figure 4e). Poly([3C]) has a modulus of 0.31 and a strength of 0.75 MPa with a strain‐at‐break of 170%. In addition, poly([3C]) also displays a strain‐hardening behavior that relating to the semicrystalline features. Poly([4C]) had similar mechanical properties; with a modulus of 0.95 MPa, and a strength of 0.29 MPa, and a strain‐at‐break of 38%. To benchmark how the mechanical properties are influenced by the dimerized monomer component, we produced a pure bath of [2C] toward poly([2C]) and assessed its properties. Compared to the previous poly([2C]), the mechanical properties are similar with a tensile strength of 18 MPa strain at break of 5.6%, and E = 630 MPa; for more information, see Figure 35, Supporting Information. However, the *T*_g_ was 20 °C higher for the pure poly([2C]), see Figure 34, Supporting Information, indicating that random inclusions of dimeric units increase the free volume of the polymer.

To assess the potential of these novel poly(β‐hydroxy acrylates) as sustainable alternatives, we compared their thermal, mechanical, and optical properties to those of conventional petroleum‐derived and bio‐based polymers. The glass transition temperatures (*T*_g_) of our materials, ranging from –29 to 46 °C, are slightly higher than those of poly(n‐butyl acrylate) (*T*_g_ ≈ −54 °C)^[^ ^52^ ^]^ and significantly lower than that of poly(methyl methacrylate) (*T*_g_ ≈ 105 °C) or polystyrene (*T*_g_ ≈ 100 °C). Mechanical properties such as tensile strength (≈18–19 MPa) and modulus (≈620–630 MPa) place our polymers between soft acrylate^[^ ^52^, ^53^ ^]^ and more rigid materials like polymethyl methacrylate (PMMA).^[^ ^54^, ^55^ ^]^ Optical transmittance (>90% in the visible range) is also on par with transparent materials such as PMMA and limonene‐based polycarbonates.^[^ ^56^, ^57^ ^]^ These comparisons highlight the potential of our system as a viable, renewable platform for a wide spectra of different polymer applications.

### 3.5 Controlled Polymerization

**Figure 7:**
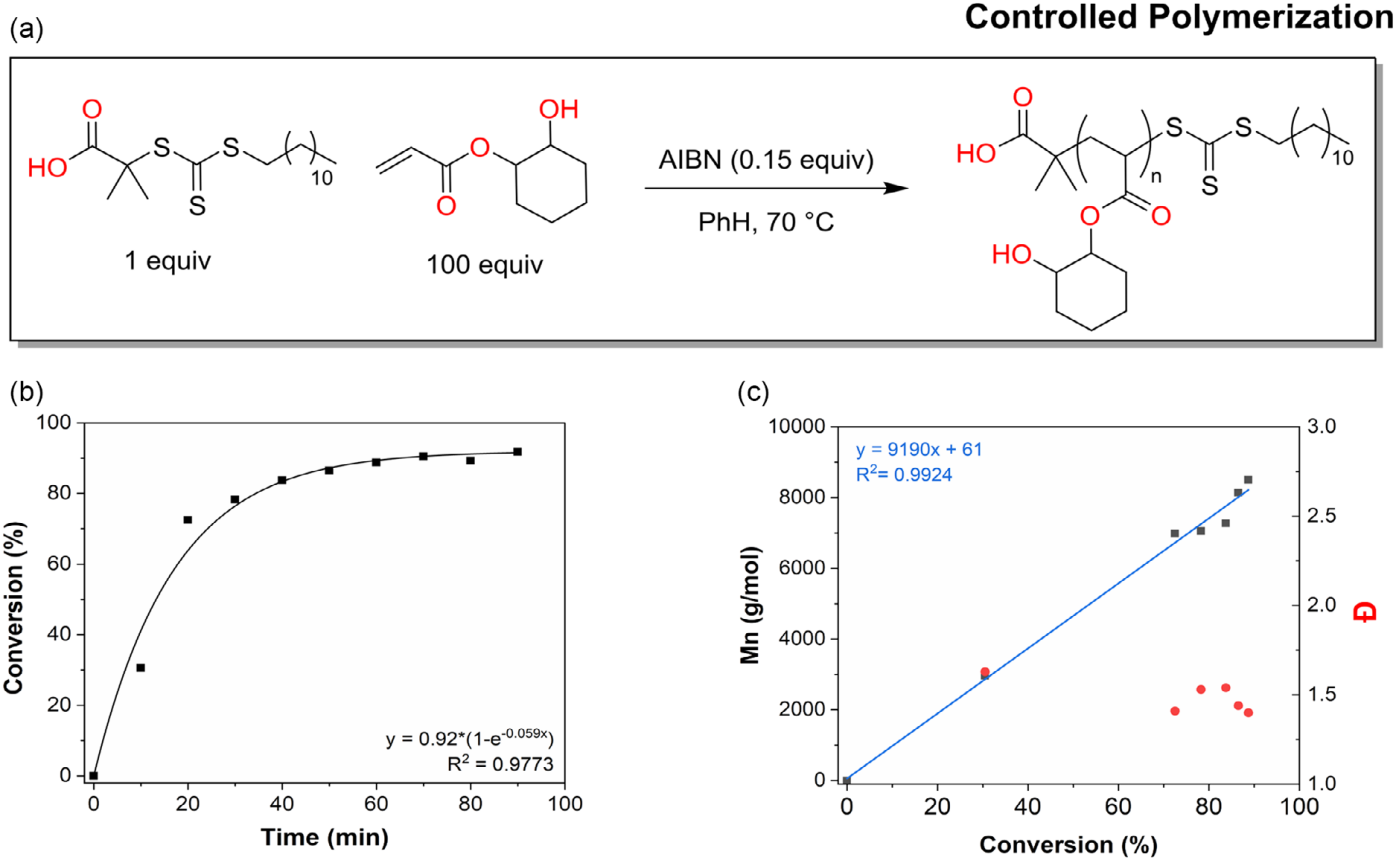
Thermally initiated RAFT polymerization. a) Reaction scheme. b) Conversion versus time, determined by ^1^ H NMR with DMF as internal standard and c) Mn against conversion, Mn was determined via SEC in CHCl_3_ using polystyrene standard.

To demonstrate the potential of β‐hydroxy acrylate monomers in precision polymer synthesis, we attempted RAFT polymerization. However, we were unable to achieve sufficient control when using the crude β‐hydroxy acrylate mixture. Instead, we needed to resort to a highly purified CO acrylate monomer [2C]; details of the purification are provided in Section S5, Supporting Information. Polymerization of [2C] was carried out using AIBN as a thermal radical initiator and DDMAT as the RAFT agent at 70 °C in toluene (Figure 6a).^[^ ^58^ ^]^ The reaction progress was monitored by ^1^H NMR to determine monomer conversion and by SEC to assess molecular weight evolution. A linear increase in *M*_n_ with conversion was observed, with Đ ≈ 1.5, indicating a more controlled polymerization process compared to previous FRP (Figure 6b,c). Furthermore, the plot of *M*_n_ versus conversion exhibited satisfactory linearity and the measured M_n_ follows the theoretical values based on conversion. These results confirm that, upon purification, [2C] can be polymerized under RAFT conditions to yield well‐defined materials suitable for more high‐end applications (**Figure** **7**).

## 4 Conclusions

Toward the next generation of more sustainable materials, polymer solutions based on abundant chemical precursors that are scalable and have the potential to redirect the chemical industry are needed. In this work, we explore AA as an oxirane nucleophile toward the formation of β‐hydroxy acrylates monomers. Four different oxiranes were explored: cyclohexane oxide, LO, ED, and EB. The different monomers were acquired in moderate to high isolated yield (68%–87%), and the reactions was performed under neat conditions. A significant excess of AA (4 equiv.) resulted in short reaction times and the optimum conditions in terms of both EMY and E‐factor of the synthesis. Unreacted AA could be re‐isolated with the help of distillation at a yield above 90%. All subsequent polymers were optically transparent with high transmittance (>91% at 550 nm) and low haze (<7% at 550 nm). The thermal stability is above 250 °C for all the polymers, except for poly(limonene β‐hydroxy‐acrylate). The structure of the initial epoxy enables access to a wide range of polymeric properties. Poly(cyclohexane β‐hydroxy‐acrylate) and poly(limonene β‐hydroxy‐acrylate) demonstrate stiff and brittle behavior with Young's modulus above 600 MPa and tensile strength from 7 to 19 MPa, while both poly(dodecane β‐hydroxy acrylate) and poly(butane β‐hydroxy‐acrylate) are highly ductile with strain‐at‐break of 170% and 38%, respectively. Controlled polymerization was exemplified by thermally initiated RAFT polymerization of purified cyclohexane β‐hydroxy‐acrylate showed a linear dependence between Mn with conversion.

Targeting fundamental transformations for monomer synthesis that enable a wide spectrum of polymeric properties is important for a potentially more benign material economy. AA as a biobased nucleophile for oxiranes shows promise. However, the low reactivity comes with the expense of control. Still, the transformation is straightforward, scalable, and general to the explored oxiranes, however for real industrial applicability the transformation and purification needs to be further optimized. Our hope is that this synthetic pathway described within will inspire work targeted toward fully biobased polymer systems centered around AA.

## Conflict of Interest

The authors declare no conflict of interest.

## Supporting information

Supplementary Material

## Data Availability

The data that support the findings of this study are available from the corresponding author upon reasonable request.
