# Supplementary material for: Exploring Acrylic Acid as an Oxirane Nucleophile: Direct Access to Poly(β‐Hydroxy Acrylates)
Source: ChemSusChem. 2025 Jun 20;18(18):e202500575. doi: 10.1002/cssc.202500575 (PMC12456388; doi:10.1002/cssc.202500575)
Supplement: Supplementary file 1 — Supplementary Material [file CSSC-18-e202500575-s001.pdf]

Exploring Acrylic Acid as an Oxirane Nucleophile - Direct Access to Poly(β-Hydroxy Acrylates)

Céline Montanari[b,c,#], Lukas Marcos Celada[a,b#], Wenhao Zhang[a,b], Peter Olsén[a,b,c*]

# Authors contributed equally

a. Laboratory of Organic Electronics, Linköping University, Norrköping 60174, Sweden

b Department of Fibre and Polymer Technology, KTH Royal Institute of Technology, Teknikringen 56, 10044 Stockholm, Sweden

c Wallenberg Wood Science Center, Laboratory of Organic Electronics, Linköping University, Norrköping 60174, Sweden

* e-mail: peter.olsen@liu.se

Electronic Supplementary Information (ESI) available: [Experimental details, NMR and characterization]. See DOI: 10.1039/x0xx00000x

Table of Contents

1. Characterization 2

2. Equations 3

3. Calculation of Green Metrics 4

3.1 Effective Mass Yield 4

3.2 E-Factor 4

4. Substrate Scope 5

4.1. Limonene oxide – acrylate 5

4.2. Cyclohexene oxide – acrylate 9

4.3. 1,2-Epoxydodecane – acrylate 13

4.4. 1,2-Epoxybutan – acrylate 17

4. Polymerization 21

4.1. Size Exclusion Chromatography (SEC) Measurements 21

4.2. Differential scanning calorimetry (DSC) 22

4.3. Pure poly([2C]) 23

5. Purified Cyclohexene-oxide acrylate and RAFT Polymerization 25

1. Characterization

.

Nuclear Magnetic Resonance (NMR). ^{1}H,^{13}C, HMBC, and COSY were recorded at room temperature on a Bruker Avance III HD 400 MHz instrument with a BBFO probe equipped with a Z-gradient coil for structural analysis. Data were processed with MestreNova (Mestrelab Research) software using a 90° shifted square sine-bell apodization window; baseline and phase correction were applied in both directions. ^{1}H NMR DOSY spectra were obtained using a stimulated echo pulse sequence with bipolar field gradients and eddy current compensation delay (Bruker sequence ledbpgp2s). The total length of the diffusion-encoding gradient and the diffusion delay were set to 2 ms and 60 ms, respectively. Gradient strength was varied linearly in 16 increments from 1 to 50 G/cm with 16 transients accumulated at each increment. The data were processed using DOSY transform in MestReNova software.

Mechanical properties. The tensile properties of the polymers (5 mm × 5 cm) were determined using a universal tensile testing machine (Instron 5944, USA) equipped with a 500 N load cell. The tests were carried out at a temperature of 22 °C and 50% RH, with a strain rate ranging from 1 to 10% min^{-1} depending on the type of sample.

Optical Properties. Transmittance and haze of the different polyacrylate films were determined using a UV-Vis spectrophotometer (UV2550, Shimadzu) with an integrating sphere module. Wavelength range span from 250 to 800 nm. Baseline was measured in air. Both transmittance and haze measurements were carried out with a slit width of 1 nm, on samples of 2.5 cm^2 with an approximate thickness of 0.5 mm. Haze was calculated using the ASTM D1003 standard. According to the standard Haze = Td/Tt, where Td and Tt are respectively the diffuse transmittance and total transmittance. After simplification Haze can calculated from the following equation: Haze = (T4/Tt) – T3, where T4 corresponds to the transmittance measured with a light trap aligned with the sample (hole in the integrating sphere) and T3 being the same measurement without the sample in front of the light source.

Differential Scanning Calorimeter (DSC). Thermal analysis of the polymers was carried out with a differential scanning calorimeter (DSC1, Mettler Toledo, Switzerland). Samples of approximately 5 to 10 mg were placed into 100 µL aluminum pans, sealed hermetically, and tested. The samples were subjected to a first cycle composed of a cooling to - 80 °C then heated to 150 °C to delete previous thermal history, then cooled to −80 °C and reheated to 200 °C. The tests were performed with a heating rate of 10 °C/min under nitrogen atmosphere. The glass transition and melting temperatures were determined from the second cycle.

Fourier Transform Infrared Spectroscopy (FTIR). A Spectrum 100 Fourier transform infrared (FTIR) spectrometer (PerkinElmer, USA) equipped with a Golden Gate diamond ATR (Gaseby Specac Ltd, UK) was used to record the spectra of the polymers. The spectra were recorded at room temperature with a resolution of 4 cm^{-1}.

The thermogravimetric analysis (TGA) Mettler Toledo TGA/DSC1, Switzerland with a heating rate of 10 °C min^{-1} under nitrogen atmosphere. The temperature at a weight loss of 5% was taken as the onset degradation temperature.

Size Exclusion Chromatography (SEC) SEC elugrams were obtained on a Malvern GPCMAX instrument containing a PLgel 5 µm guard column (7.5 x 50 mm) and two PLgel 5 µm MIXED-D (300 x 7.5 mm) columns. HPLC grade $CHCl_3$ containing 2% (v/v) toluene was used as eluent at 35 °C. Polystyrene standards with a narrow dispersity (162-364,000 g mol^{-1}) were used as calibration. Samples were prepared at ~3 mg mL^{-1} for a 100 µL injection volume.

2. Equations

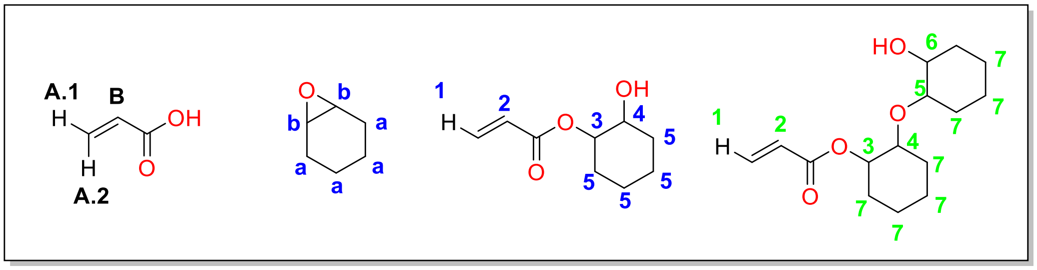

$$E.1\,(Conversion\ Acylic\ acid) = 100 * \frac{\int_{5.75\,ppm}^{5.88\,ppm}(1+1) + \int_{2.67\,ppm}^{2.79\,ppm}(\boldsymbol{ii})/2}{\int_{5.9ppm}^{6.0\,ppm}\mathbf{B} + \int_{5.75\,ppm}^{5.88\,ppm}(1+1) + \int_{2.67\,ppm}^{2.79\,ppm}(\boldsymbol{ii})/2}$$

$$E.2\,(Conversion\ Cyclohexene\ oxid) = 100 * \left(1 - \frac{\int_{3,11\,ppm}^{3.18\,ppm}\frac{\boldsymbol{b}}{\mathbf{2}}}{\frac{\int_{1.0\,ppm}^{2.4\,ppm}(a+5+7)}{8}}\right)$$

$$E.4\,(Degree\ of\ oligomerization) = \frac{\int_{4.60\,ppm}^{4.75\,ppm}(3)}{\int_{4.60\,ppm}^{4.75\,ppm}(3) + \int_{4.75\,ppm}^{4.88\,ppm}(3)}$$

3. Calculation of Green Metrics

3.1 Effective Mass Yield

Effective Mass Yield (EMY) is a combined green chemistry metric that reflects both the theoretical efficiency of a reaction and its practical execution. It is calculated as the product of atom economy and reaction yield, providing a realistic measure of how much of the reactants' mass ends up in the final product. While atom economy assesses how well a reaction incorporates the atoms of starting materials into the desired product, and yield measures the experimental success of the transformation, EMY accounts for both. A high EMY indicates that a reaction is not only conceptually efficient but also works well in practice. In this study, EMY is used to evaluate the sustainability of the ring-opening acrylation reactions.

EMY= Atom Economy × Yield

Example Reaction: Entry 10 Table 1 main manuscript

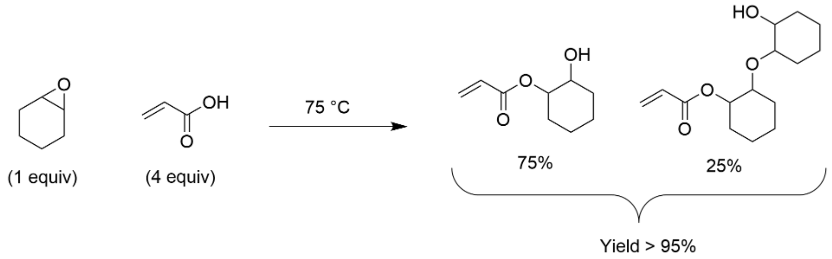

AE for this transformation is 1 since all the reactant is included in the product.

EMY = 1*0.95 = 0.95

3.2 E-Factor

The E-factor (Environmental factor) measures the environmental impact of a chemical process by quantifying the amount of waste generated per unit mass of product. It is calculated as the total mass of all waste materials (including solvents, reagents, and byproducts) divided by the mass of the desired product. Lower E-factors indicate cleaner, more sustainable processes. In this study, we used the E-factor to assess the overall material efficiency of each synthetic route, taking into account both reaction inputs and workup procedures, in order to identify strategies with reduced environmental burden.

E-factor= Total mass of waste/Total mass of desired product
Example Reaction: Entry 10 Table 1 main manuscript

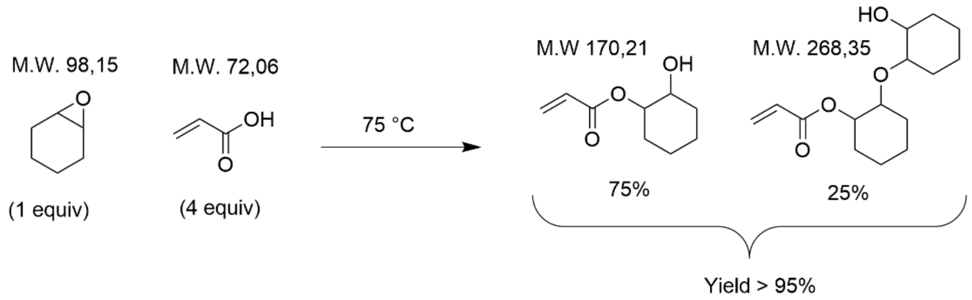

E-Factor = (0.05*(98.15+72.06) + 0.10*72.06*3)/(170.21*0.75+268.35*0.25)*0.95= 30.1/185 = 0.16

4. Substrate Scope

4.1. Limonene oxide – acrylate

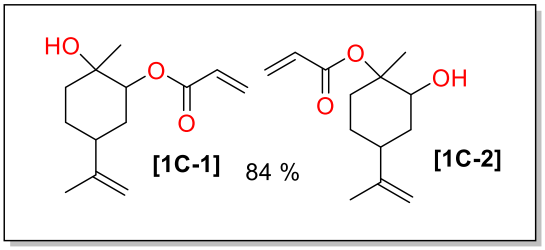

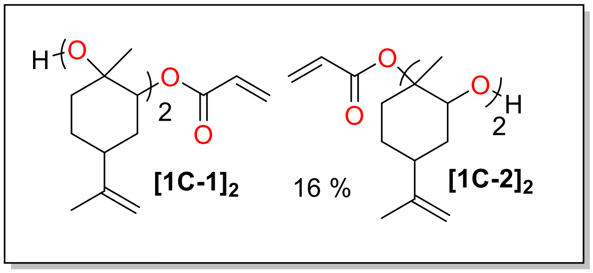

2-hydroxy-2-methyl-5-(prop-1-en-2-yl)cyclohexyl acrylate (**[1C-1]**) and 2-hydroxy-1-methyl-4-(prop-1-en-2-yl)cyclohexyl acrylate (**[1C-2]**) in accordance to previous described protocol, 33 g (yield 87 % as a transparent oil). The degree of olimerization was 1.22, and the product ratios is **[1C-1]** : **[1C-2]** = 1.0 : 0.88. ^{1}H NMR (400 MHz, Chloroform-d) δ 6.36 (ddd, J = 35.8, 17.3, 1.5 Hz, 1H), 6.10 (ddd, J = 31.3, 17.4, 10.4 Hz, 1H), 5.81 (ddd, J = 30.7, 10.3, 1.5 Hz, 1H), 4.91 (q, J = 3.2, 2.3 Hz, 0.55H), 4.73 – 4.70 (m, 2H), 4.14 (q, J = 3.5 Hz, 0.45H), 2.39 – 1.10 (m, 13H). ^{13}C NMR (101 MHz, Chloroform-d) δ 165.34 , 165.20 , 149.22 , 149.03 , 130.79 , 130.03 , 129.88 , 129.02 , 128.72 , 128.21 , 109.08 , 108.94 , 83.07 , 75.49 , 70.45 , 69.87 , 38.16 , 36.99 , 34.50 , 33.77 , 31.05 , 30.51 , 27.06 , 26.08 , 25.99 , 21.68 , 20.99 , 20.92 .

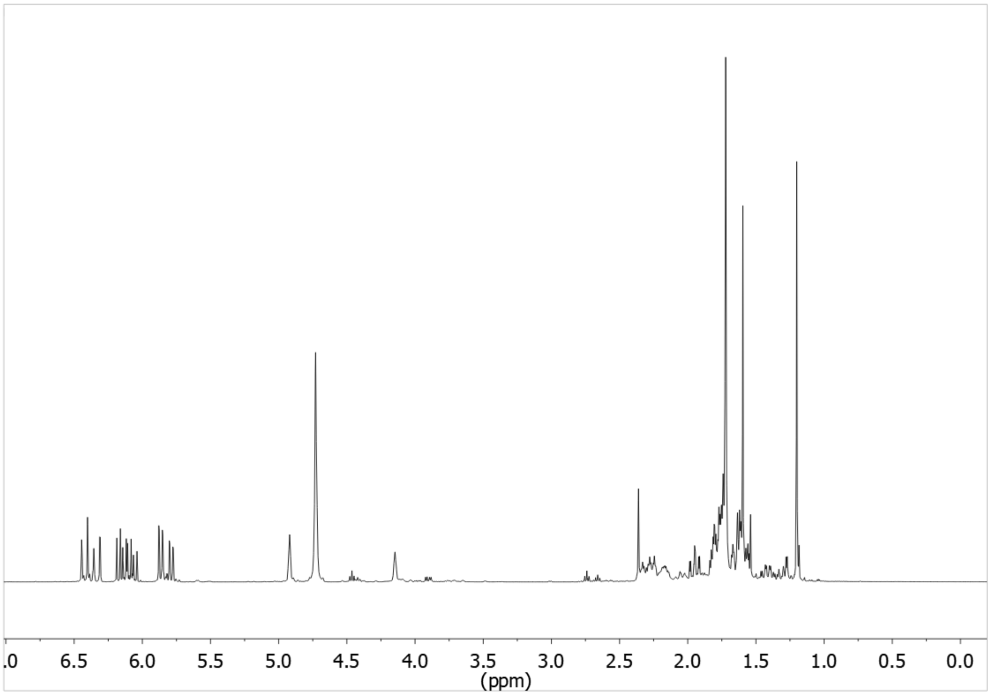

Figure S1. ^{1}H NMR spectrum (400 MHz, $CDCl_3$) of 2-hydroxy-2-methyl-5-(prop-1-en-2-yl)cyclohexyl acrylate and 2-hydroxy-1-methyl-4-(prop-1-en-2-yl)cyclohexyl acrylate. Acrylate vinyl protons appear at δ 6.36, 6.10, and 5.81 ppm. The isopropenyl methylene group (CH_2=C) resonates at δ 4.73–4.70 ppm. Hydroxyl-bearing methines are observed at δ 4.91 and 4.14 ppm. Aliphatic ring and side-chain protons resonate from δ 2.39 to 1.10 ppm.

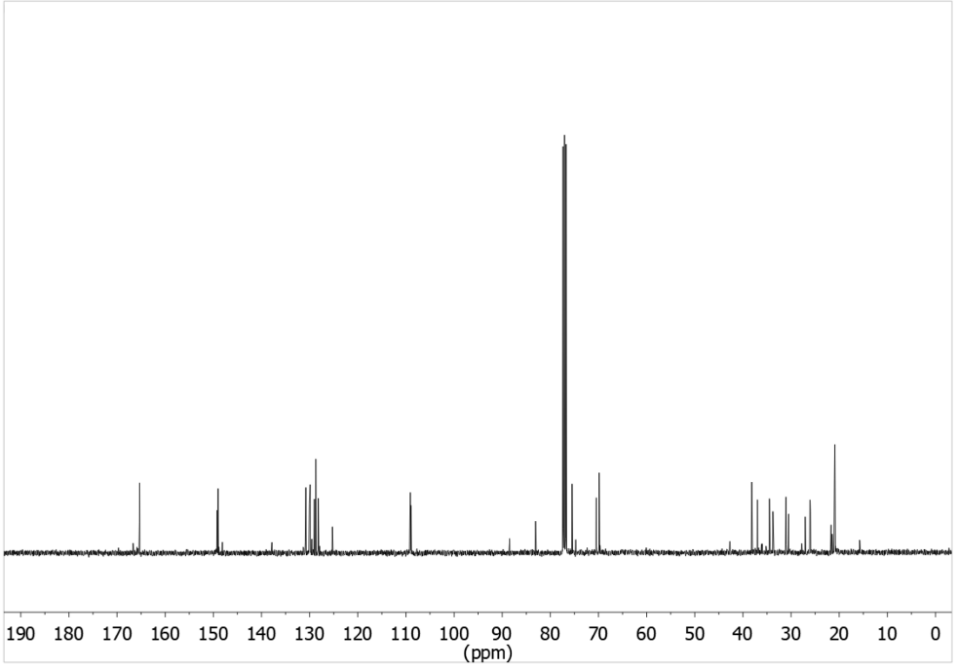

Figure S2. ^{13}C NMR spectrum (101 MHz, $CDCl_3$) of 2-hydroxy-2-methyl-5-(prop-1-en-2-yl)cyclohexyl acrylate and 2-hydroxy-1-methyl-4-(prop-1-en-2-yl)cyclohexyl acrylate. Acrylate carbonyl carbons appear at δ ~165 ppm, vinyl and isopropenyl carbons between δ 149–128 ppm, isopropenyl quaternary carbon at δ ~109 ppm, oxygenated methines and methylenes between δ 83–69 ppm, and aliphatic cycloalkyl and methyl carbons from δ 38 to 20 ppm.

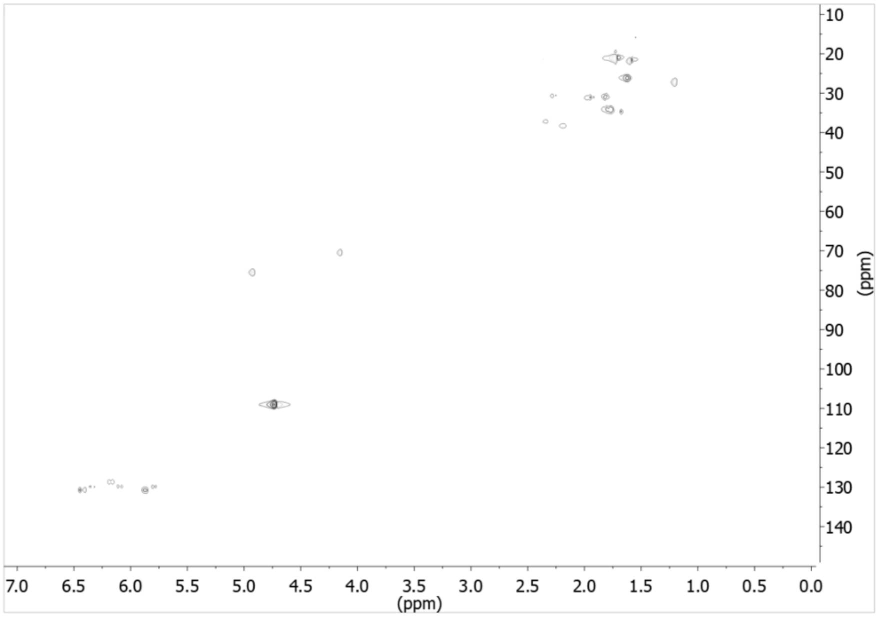

Figure S3. HSQC spectrum of 2-hydroxy-2-methyl-5-(prop-1-en-2-yl)cyclohexyl acrylate and 2-hydroxy-1-methyl-4-(prop-1-en-2-yl)cyclohexyl acrylate in $CDCl_3$. Clear ^1H–^{13}C correlations confirm assignments of acrylate vinyl protons (δH 6.36–5.81, δC ~130–128 ppm), isopropenyl CH_2 group (δH ~4.7, δC ~109 ppm), hydroxyl-bearing methines (δH ~4.9 and 4.1, δC ~75–70 ppm), and multiple aliphatic CH, CH_2 and CH_3 groups (δH 2.4–1.1, δC 38–20 ppm).

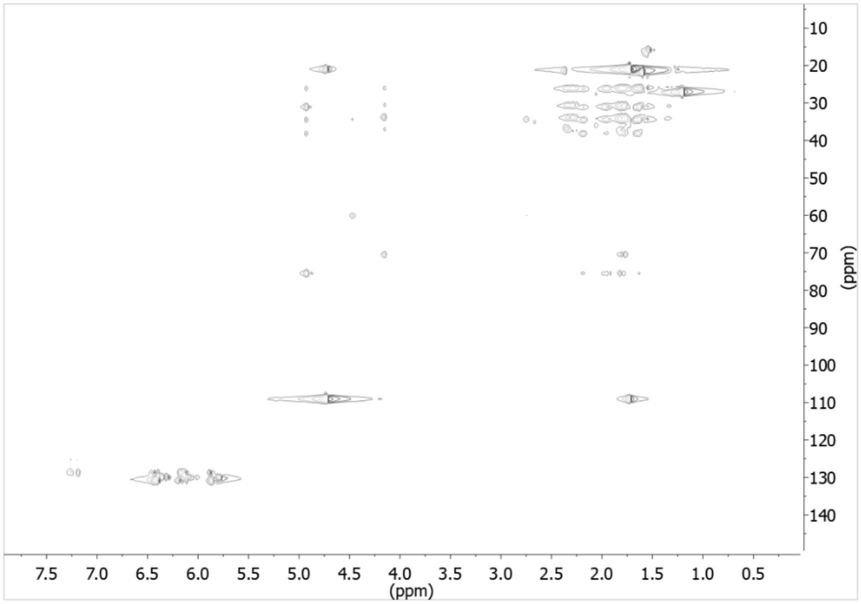

Figure S4. HMBC spectrum of 2-hydroxy-2-methyl-5-(prop-1-en-2-yl)cyclohexyl acrylate and 2-hydroxy-1-methyl-4-(prop-1-en-2-yl)cyclohexyl acrylate in $CDCl_3$. Key long-range ^1H–^{13}C correlations are observed between the vinylic protons of the acrylate moiety (δH 6.36–5.81 ppm) and the adjacent sp^2 carbon signals (~128–130 ppm), supporting acrylate substitution. Correlations from hydroxyl-bearing methines (δH ~4.9 and ~4.1 ppm) to neighboring aliphatic and oxygenated carbons help establish the ring substitution pattern and distinguish between the isomers.

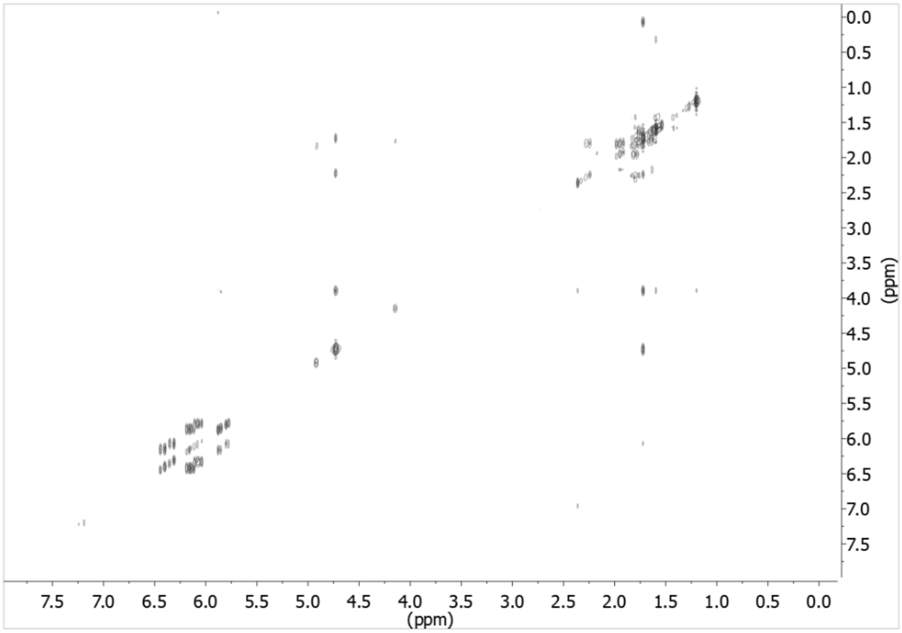

Figure S5. ^1H–^1H COSY spectrum of 2-hydroxy-2-methyl-5-(prop-1-en-2-yl)cyclohexyl acrylate and 2-hydroxy-1-methyl-4-(prop-1-en-2-yl)cyclohexyl acrylate. Correlation peaks confirm coupling between vinylic protons, hydroxyl-bearing methines, and adjacent aliphatic protons, aiding full proton network assignment.

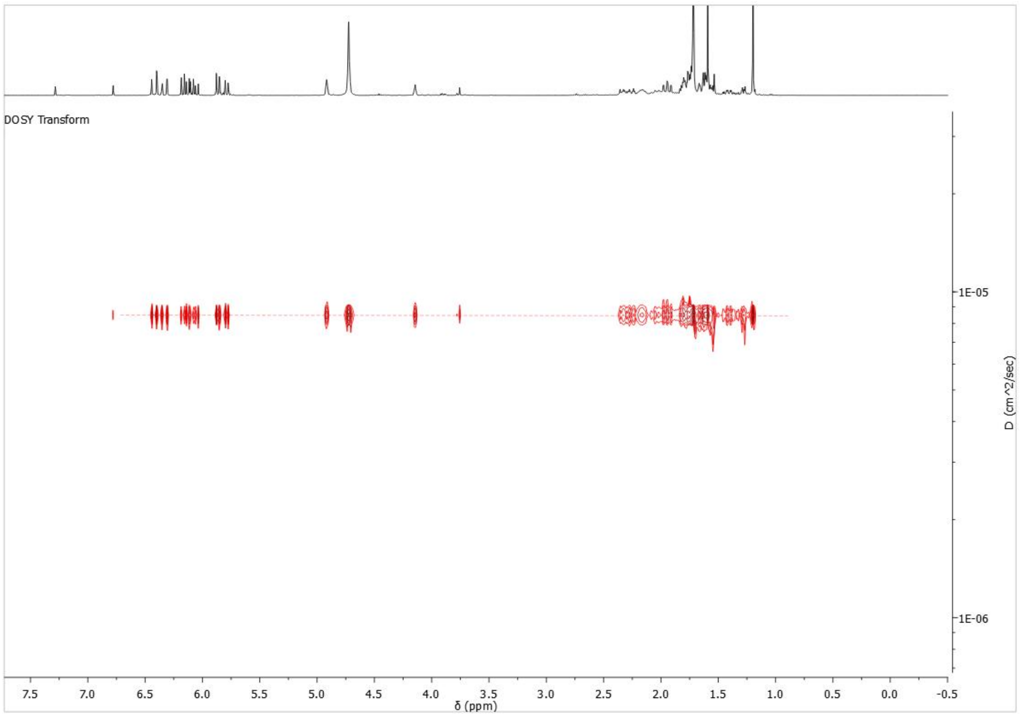

Figure S6. DOSY spectrum of the limonene-derived acrylate mixture. The diffusion coefficients indicate the presence of distinct molecular species. Closely grouped diffusion signals suggest monomeric forms, while slight spreading indicate oligomeric content.

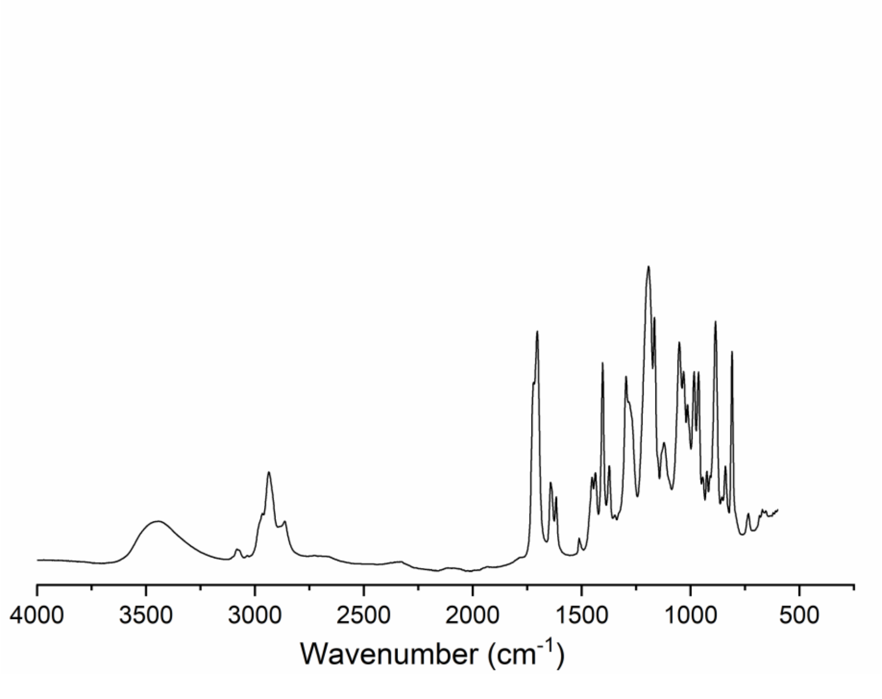

Figure S7. FTIR spectrum of 2-hydroxy-2-methyl-5-(prop-1-en-2-yl)cyclohexyl acrylate and 2-hydroxy-1-methyl-4-(prop-1-en-2-yl)cyclohexyl acrylate. Characteristic absorptions are observed for acrylate C=O stretching (~1720 cm^{-1}), C=C stretching (~1635 cm^{-1}), and broad O–H stretching (~3400 cm^{-1}), confirming the presence of hydroxyl and acrylate functionalities.

4.2. Cyclohexene oxide – acrylate

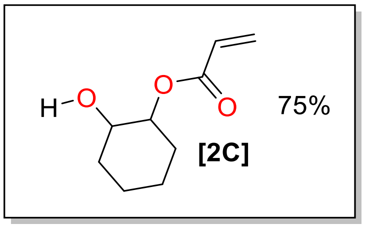

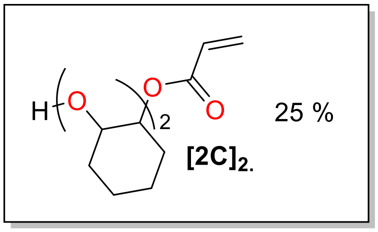

2-hydroxycyclohexyl acrylate (**[2C]**) Cis and Trans, in accordance to previous described protocol, 26 g (yield 84 % as a transparent oil). The degree of olimerization was 1.43 ^{1}H NMR (400 MHz, Chloroform-d) δ 6.40 (dd, J = 17.3, 1.5 Hz, 1H), 6.12 (dd, J = 17.3, 10.4 Hz, 1H), 5.82 (dd, J = 10.4, 1.5 Hz, 1H), 4.77 (dtd, J = 9.9, 8.6, 4.6 Hz, 0.3H), 4.64 (ddt, J = 9.0, 5.8, 4.6 Hz, 0.7H), 3.59 (ddd, J = 10.7, 8.9, 4.6 Hz, 0.7H), 3.40 – 3.26 (m, 0.3H), 2.12 – 1.91 (m, 2H), 1.76 – 1.61 (m, 2H), 1.37 – 1.13 (m, 2H). ^{13}C NMR (101 MHz, Chloroform-d) δ 166.32 , 130.94 , 128.59 , 78.29 , 72.58 , 32.96 , 29.90 , 23.83 , 23.72 .

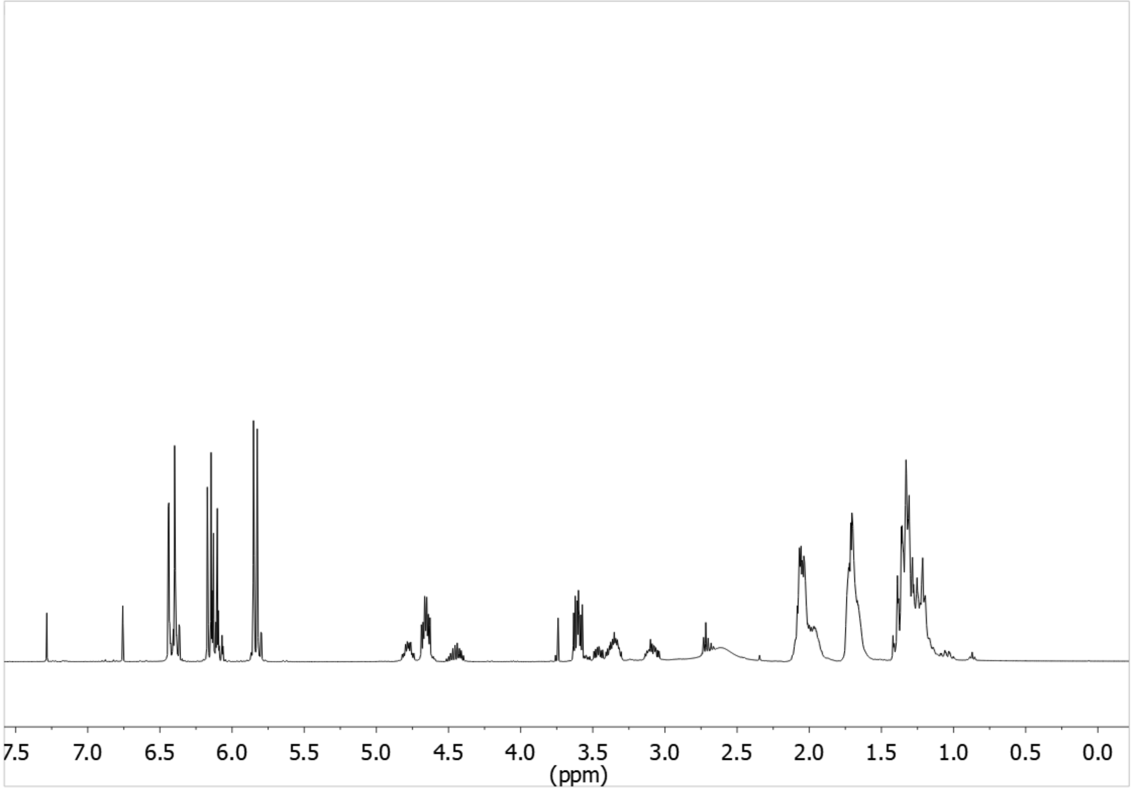

Figure S8. ^{1}H NMR spectrum (400 MHz, $CDCl_3$) of 2-hydroxycyclohexyl acrylate ([2C], cis and trans isomers). Acrylate vinyl protons are clearly resolved at δ 6.40, 6.12, and 5.82 ppm. The hydroxyl-bearing methines appear as split signals between δ 4.77 and 3.26 ppm due to the presence of cis/trans isomers. Cyclohexyl ring methylenes give rise to multiplets in the region δ 2.12–1.13 ppm. Additional minor signals consistent with dimeric species—such as downfield-shifted methines and broadened multiplets in the aliphatic region—can also be observed, showing the dimerization.

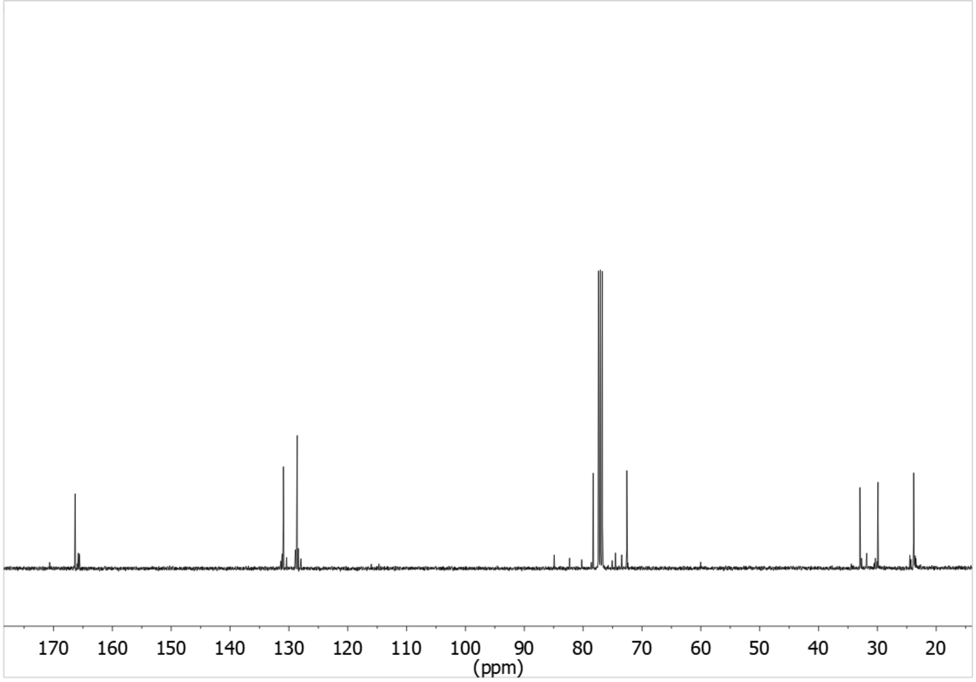

Figure S9. 13C NMR spectrum (101 MHz, $CDCl_3$) of 2-hydroxycyclohexyl acrylate. The acrylate carbonyl and vinyl carbons resonate at δ 166.32, 130.94, and 128.59 ppm. The two oxygenated carbons in the ring (bearing OH and attached to acrylate) appear at δ 78.29 and 72.58 ppm. Signals from aliphatic ring carbons span δ 32.96 to 23.72 ppm.

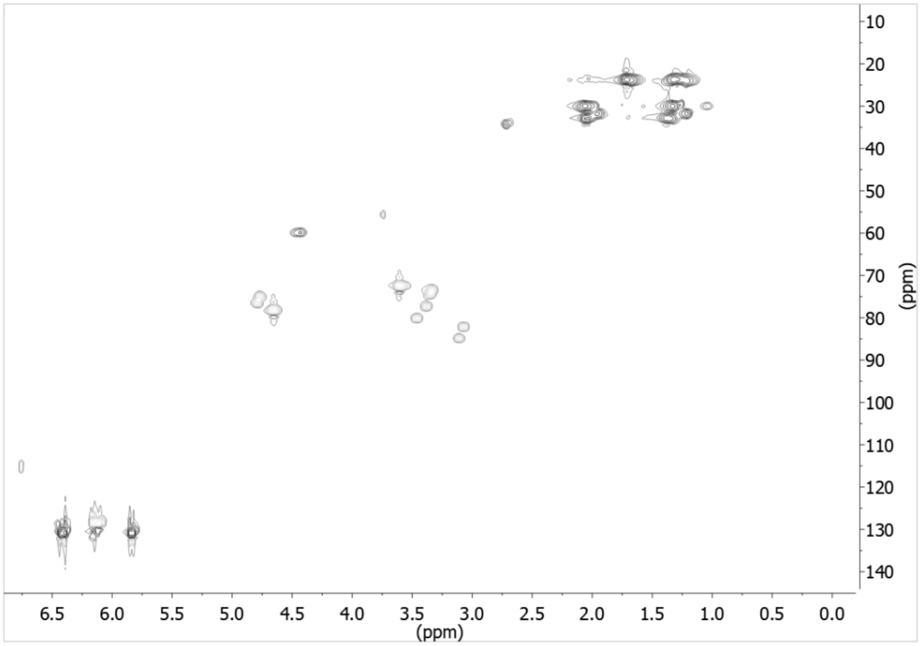

Figure S10. HSQC spectrum of 2-hydroxycyclohexyl acrylate. Direct ^{1}H–^{13}C correlations confirm assignments for the acrylate vinyl protons and carbons (δH 6.40–5.82 ppm; δC ~130–128 ppm), hydroxyl-bearing methines (δH 4.77–3.26 ppm; δC ~78–72 ppm), and cyclohexyl ring methylenes (δH 2.1–1.1 ppm; δC ~33–24 ppm).

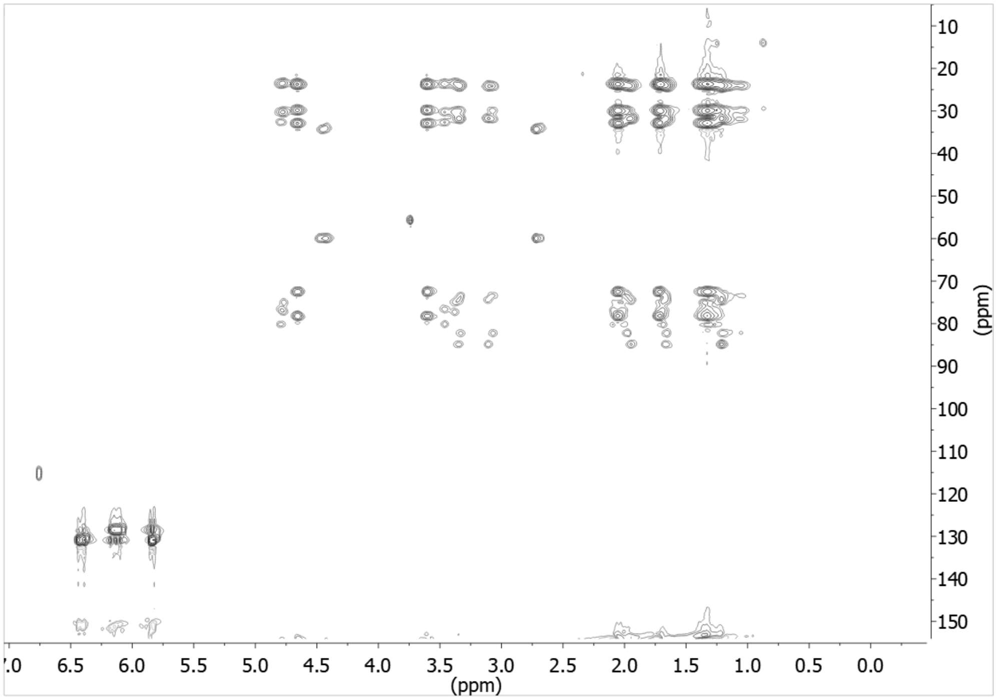

Figure S11. HMBC spectrum of 2-hydroxycyclohexyl acrylate. Long-range ^{1}H–^{13}C correlations between acrylate vinyl protons and adjacent sp² carbons support the acrylate structure.

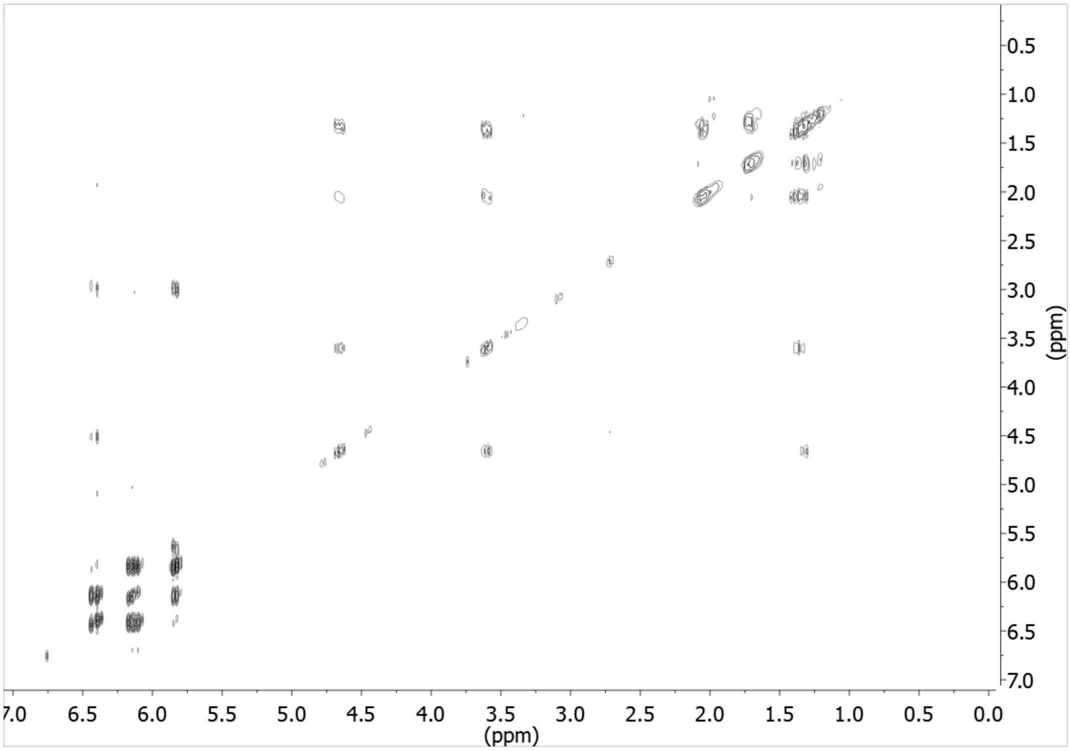

Figure S12. ^{1}H–^{1}H COSY spectrum of 2-hydroxycyclohexyl acrylate. Coupling patterns confirm the spin systems within the cyclohexyl ring. Cross-peaks between the hydroxyl methines and adjacent methylene groups help distinguish between cis and trans isomers.

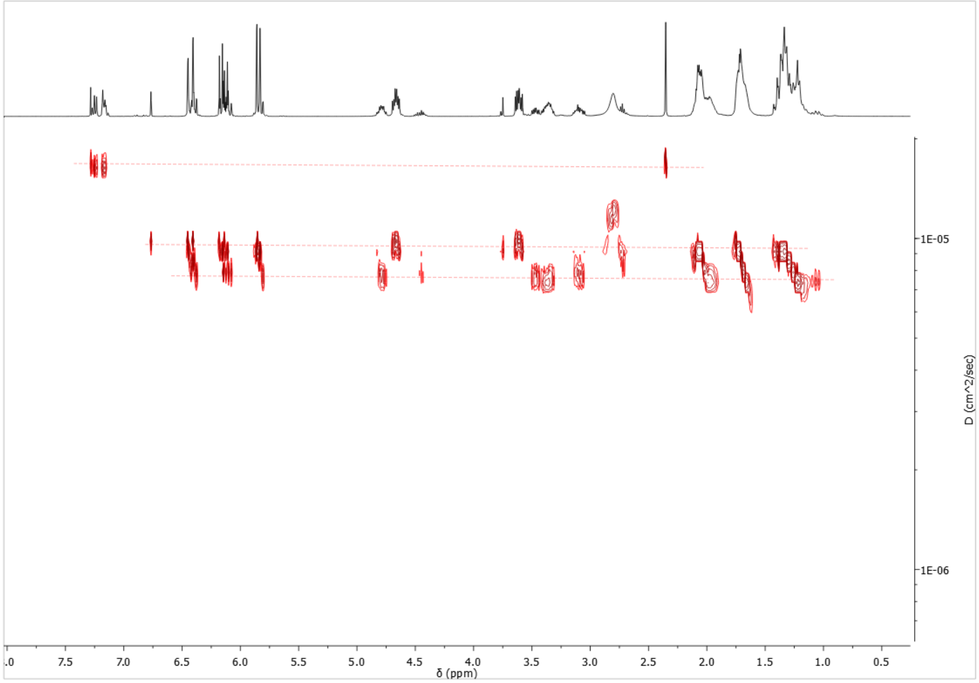

Figure S13. DOSY spectrum of 2-hydroxycyclohexyl acrylate. A single diffusion coefficient dominates the spectrum, indicating that the sample consists primarily of a mono and dimeric species. No evidence of higher oligomers is detected, consistent with NMR integration.

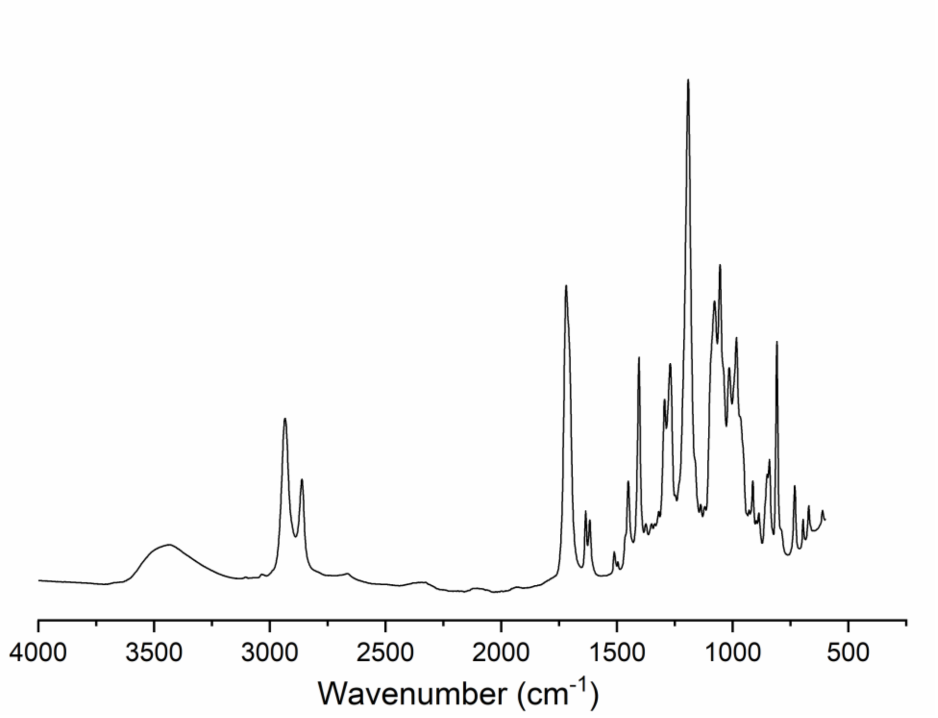

Figure S14. FTIR spectrum of 2-hydroxycyclohexyl acrylate. A strong absorption at ~1720 cm^{-1} confirms the presence of the acrylate ester carbonyl. C=C stretching appears around 1635 cm^{-1}, while a broad O–H stretch centered around 3400 cm^{-1} is consistent with free hydroxyl functionality.

4.3. 1,2-Epoxydodecane – acrylate

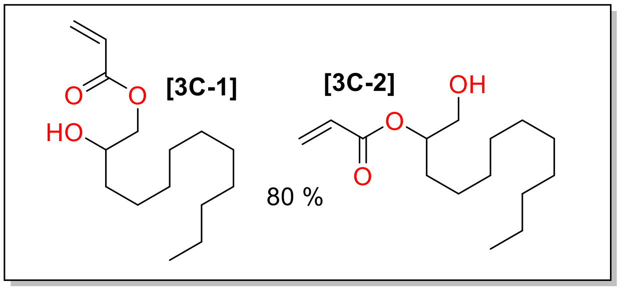

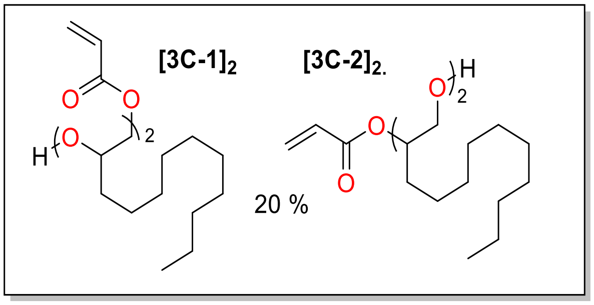

2-hydroxydecyl acrylate (**[3C-1]**) and 1-hydroxydecan-2-yl acrylate (**[3C-2]**). The reaction was performed in accordance to previous described protocol except that the reaction time was 24 h, 26 g (yield 86 % as a transparent oil). The degree of olimerization was 1.43, and the product ratios is **[3C-1]** : **[3C-2]** = 1.0 : 0.59. ^{1}H NMR (400 MHz, Chloroform-d) δ 6.52 – 6.35 (m, 1H), 6.15 (ddd, J = 17.3, 10.5, 5.0 Hz, 1H), 5.85 (ddd, J = 10.4, 4.7, 1.5 Hz, 1H), 5.15 – 4.91 (m, 0.4H), 4.23 (dd, J = 11.4, 3.1 Hz, 0.6H), 4.05 (dd, J = 11.5, 7.3 Hz, 0.6H), 3.88 (qd, J = 6.9, 6.4, 3.1 Hz, 0.6H), 3.79 – 3.58 (m, 0.8H), 2.32 (s, 1H), 1.69 – 1.16 (m, 15H), 0.88 (t, J = 6.6 Hz, 3H). ^{13}C NMR (101 MHz, Chloroform-d) δ 166.38 (d, J = 18.8 Hz), 131.12 (d, J = 22.7 Hz), 128.28 (d, J = 40.4 Hz), 75.87 , 69.90 , 68.80 , 64.63 , 33.36 , 31.88 , 29.83 – 28.97 (m), 25.32 (d, J = 9.6 Hz), 22.66 , 14.08 .

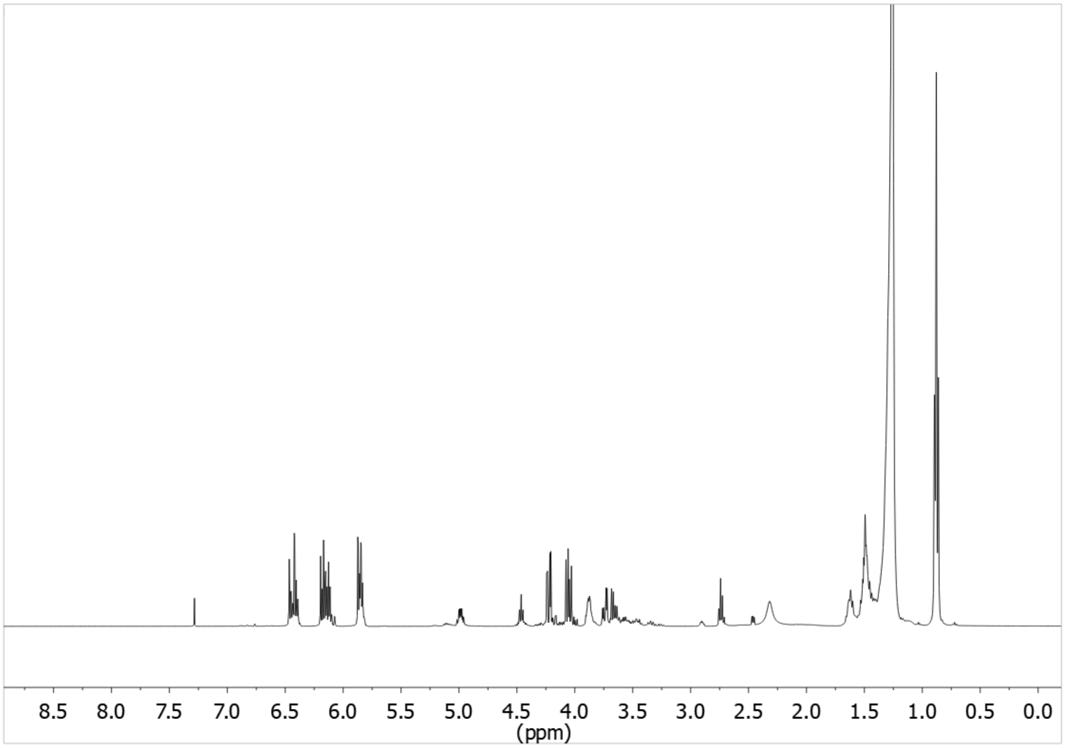

Figure S15. ^{1}H NMR spectrum (400 MHz, $CDCl_3$) of 2-hydroxydecyl acrylate ([3C-1]) and 1-hydroxydecan-2-yl acrylate ([3C-2]). Acrylate vinyl protons are observed between δ 6.52–5.85 ppm. Signals at δ 5.15–3.58 ppm correspond to hydroxyl-bearing methine and methylene groups from both regioisomers. The long aliphatic chain produces a complex multiplet between δ 1.69–1.16 ppm and a terminal methyl triplet at δ 0.88 ppm.

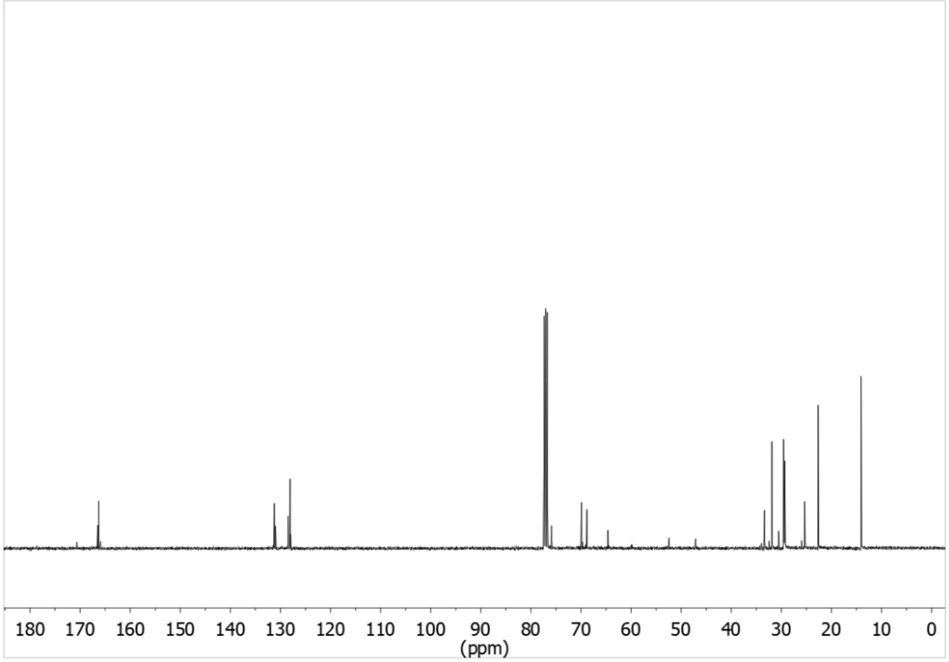

Figure S16. ^{13}C NMR spectrum (101 MHz, $CDCl_3$) of 2-hydroxydecyl acrylate and 1-hydroxydecan-2-yl acrylate. The acrylate carbonyl and vinyl carbons appear at δ 166.38, 131.12, and 128.28 ppm. Oxygenated carbons resonate at δ 75.87, 69.90, 68.80, and 64.63 ppm, while the decyl chain gives characteristic signals between δ 33.36 and 14.08 ppm.

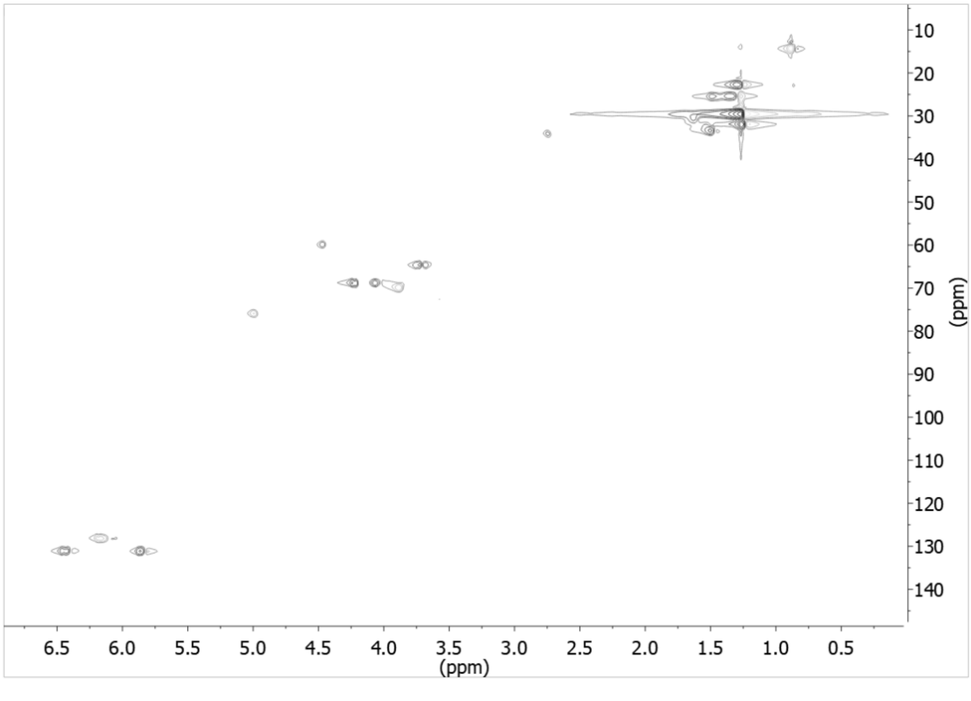

Figure S17. HSQC spectrum of 2-hydroxydecyl acrylate and 1-hydroxydecan-2-yl acrylate. Correlations confirm vinyl CH=CH2 assignments (δH 6.52–5.85 ppm, δC 131–128 ppm), along with direct ^1H–^{13}C pairs for hydroxyl-bearing methines and methylenes in the δH 5.15–3.58 ppm / δC 76–64 ppm region. Multiple CH2 signals confirm a long alkyl chain.

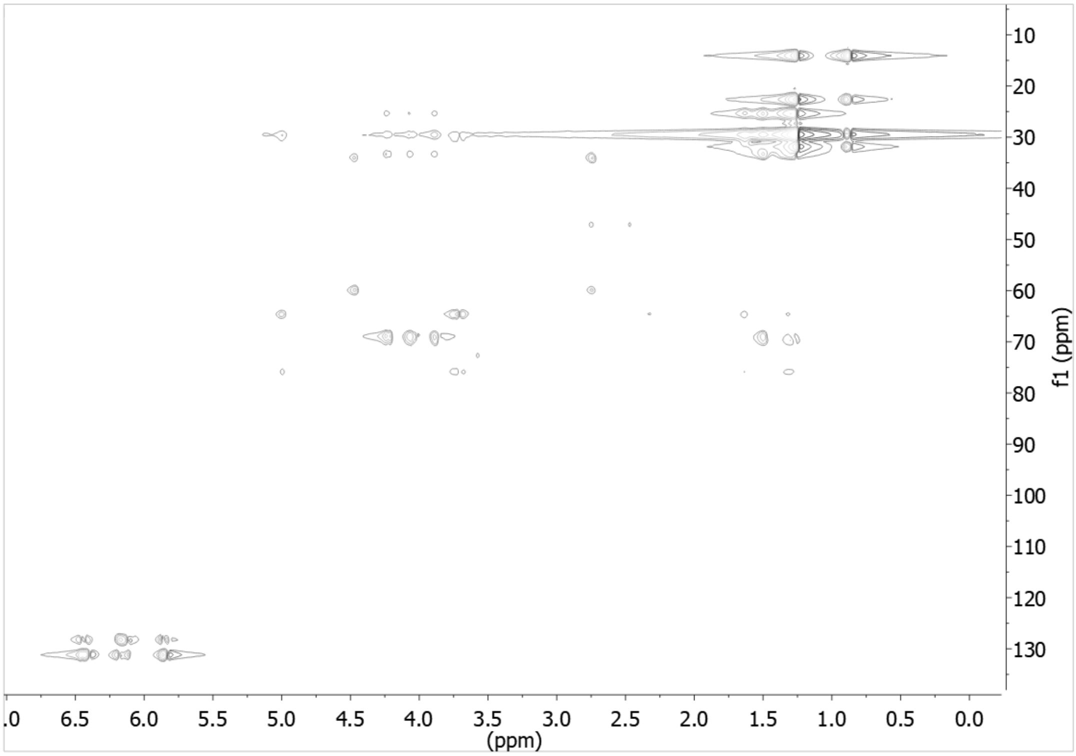

Figure S18. HMBC spectrum of 2-hydroxydecyl acrylate and 1-hydroxydecan-2-yl acrylate. Long-range ^{1}H–^{13}C correlations support the assignment of the acrylate moiety, hydroxyl-bearing carbons.

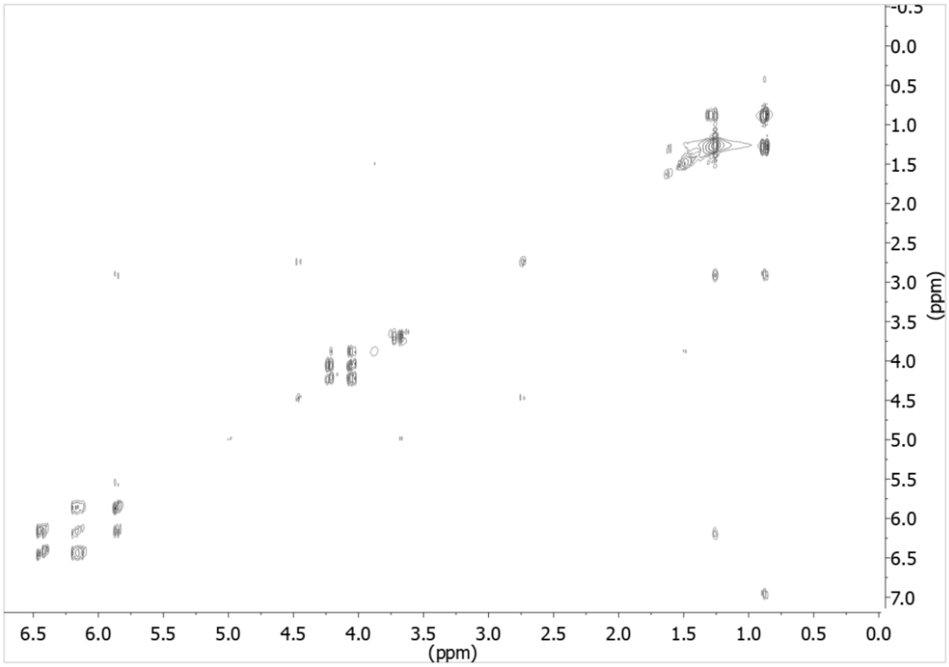

Figure S19. ^{1}H–^{1}H COSY spectrum of 2-hydroxydecyl acrylate and 1-hydroxydecan-2-yl acrylate. Key coupling patterns among the hydroxyl methines, methylene groups, and terminal methyl protons aid in confirming the proton network and differentiate the two isomers.

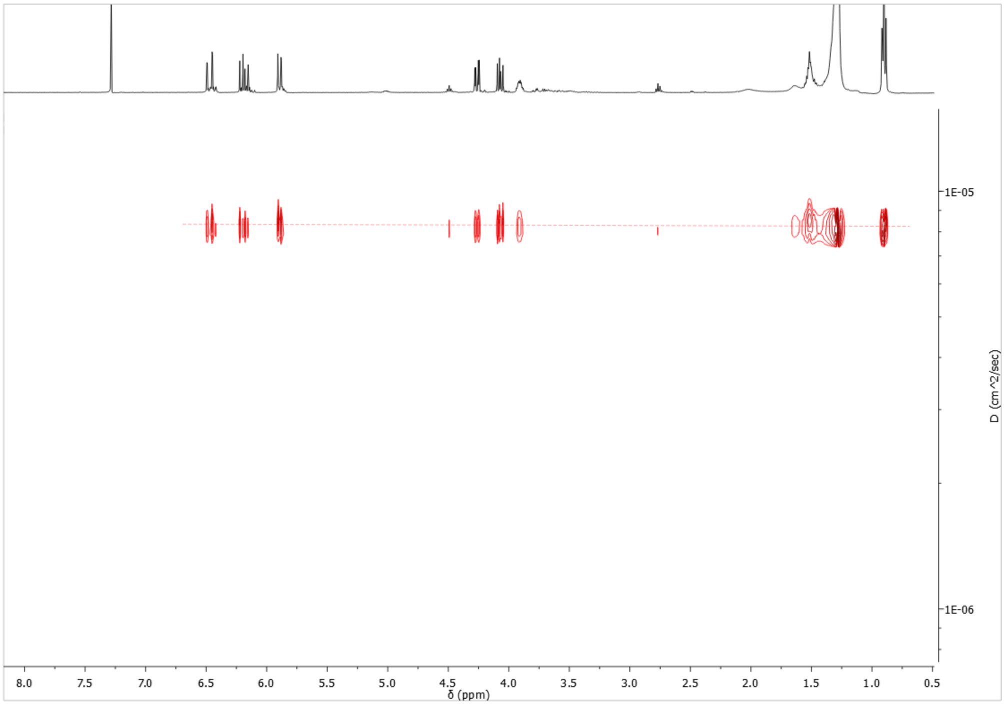

Figure S20. DOSY spectrum of 2-hydroxydecyl acrylate and 1-hydroxydecan-2-yl acrylate. Single diffusion coefficient observed for each product supports sample homogeneity and purity, indicating absence of significant oligomeric or polymeric species.

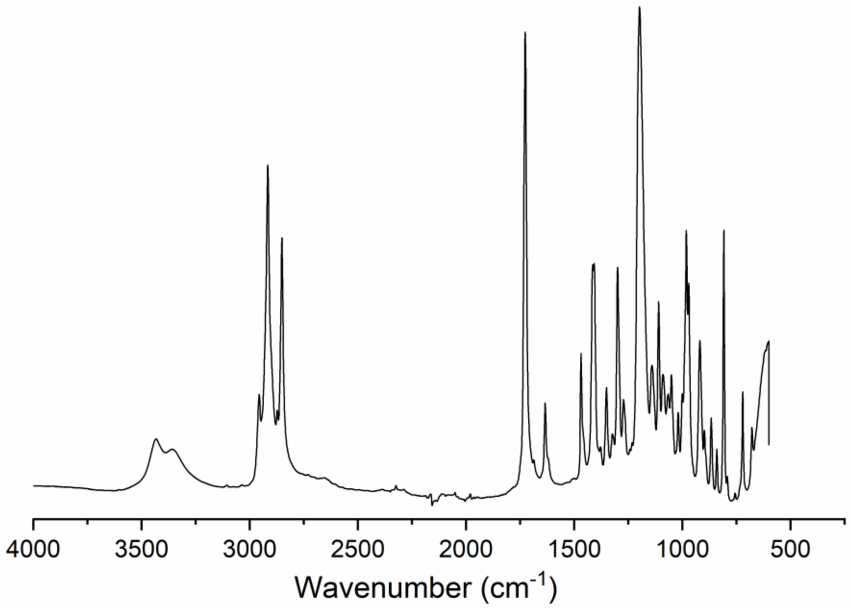

Figure S21. FTIR spectra of 2-hydroxydecyl acrylate and 1-hydroxydecan-2-yl acrylate. Characteristic absorption bands include broad O–H stretching (~3400 cm^{-1}), C=O stretching (~1720 cm^{-1}), and C–O stretching (1150–1050 cm^{-1}), confirming acrylate ester and hydroxyl functionality.

4.4. 1,2-Epoxybutan – acrylate

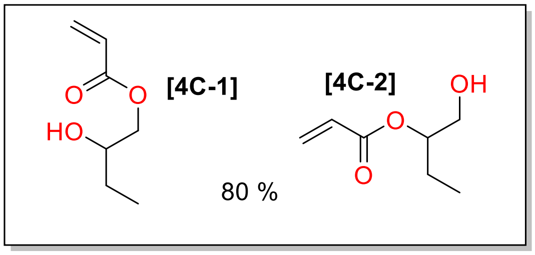

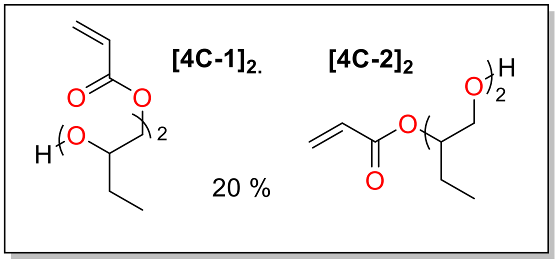

2-hydroxybutyl acrylate (**[4C-1]**) and 1-hydroxybutan-2-yl acrylate (**[4C-2]**). The reaction was performed in accordance to previous described protocol except that the reaction time was 80 h and the temperature was 60 °C, 41g (yield 68 % as a transparent oil). The degree of olimerization was 1.26, and the product ratios is **[4C-1]** : **[4C-2]** = 1.0 : 0.57. ^{1}H NMR (400 MHz, Chloroform-d) δ 6.43 – 6.30 (m, 1H), 6.16 – 6.03 (m, 1H), 5.84 – 5.75 (m, 1H), 5.07 – 4.79 (m, 0.36H), 4.16 (dd, J = 11.4, 3.4 Hz, 1.32H), 4.02 (dd, J = 11.4, 7.0 Hz, 1.32H), 3.80 – 3.60 (m, 2.04H), 1.66 – 1.35 (m, 2H), 0.98 – 0.81 (m, 3H). ^{13}C NMR (101 MHz, Chloroform-d) δ 170.78, 170.69 , 166.39 , 166.28 , 131.01 (d, J = 26.5 Hz), 128.28 (d, J = 40.2 Hz), 76.81 , 70.89 (d, J = 16.7 Hz), 68.43 (d, J = 20.7 Hz), 63.84, 26.33 , 23.53 , 11.16 – 7.43 (m).

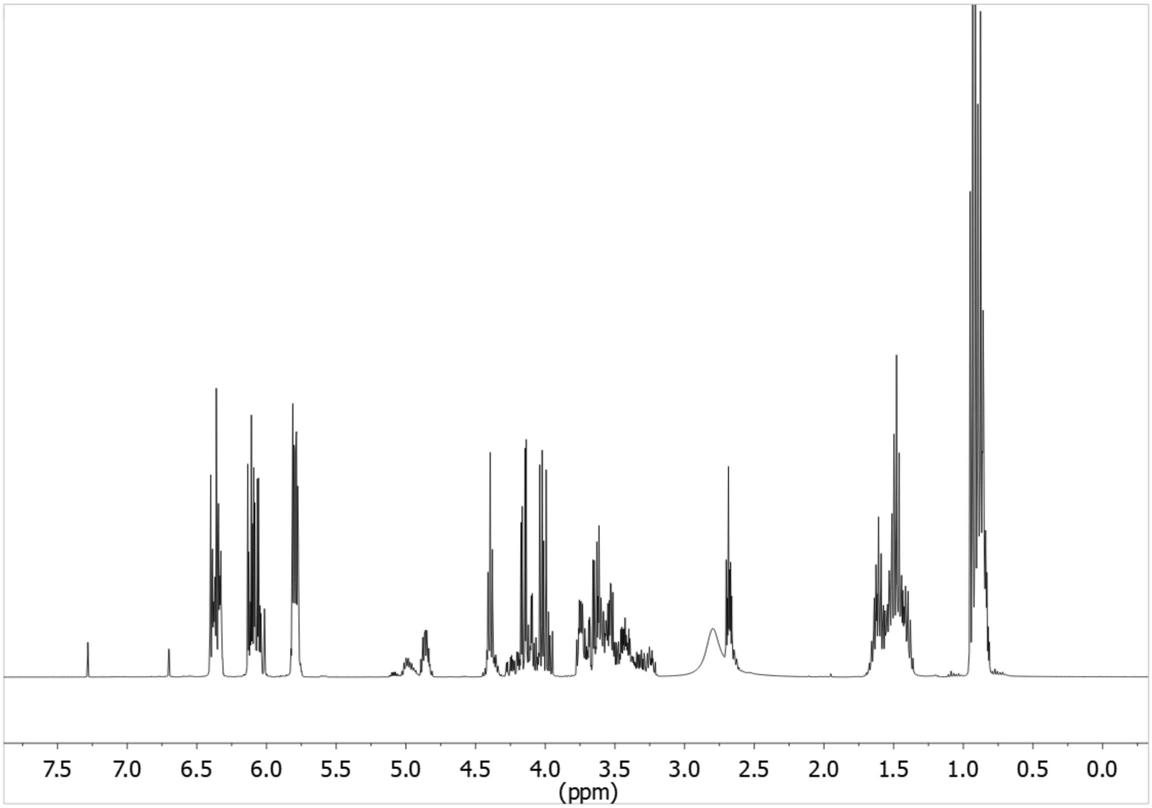

Figure S22. ^{1}H NMR spectrum (400 MHz, $CDCl_3$) of hydroxybutyl acrylate ([4C-1]) and 1-hydroxybutan-2-yl acrylate ([4C-2]). The acrylate vinyl protons are observed at δ 6.43–5.75 ppm as multiplets. The hydroxyl-bearing methylene and methine groups resonate at δ 5.07–3.40 ppm, reflecting differences between the linear and branched isomers. Aliphatic methylene and methyl groups appear at δ 1.66–0.81 ppm. The integrals confirm a [4C-1] : [4C-2] ratio of 1.0 : 0.57.

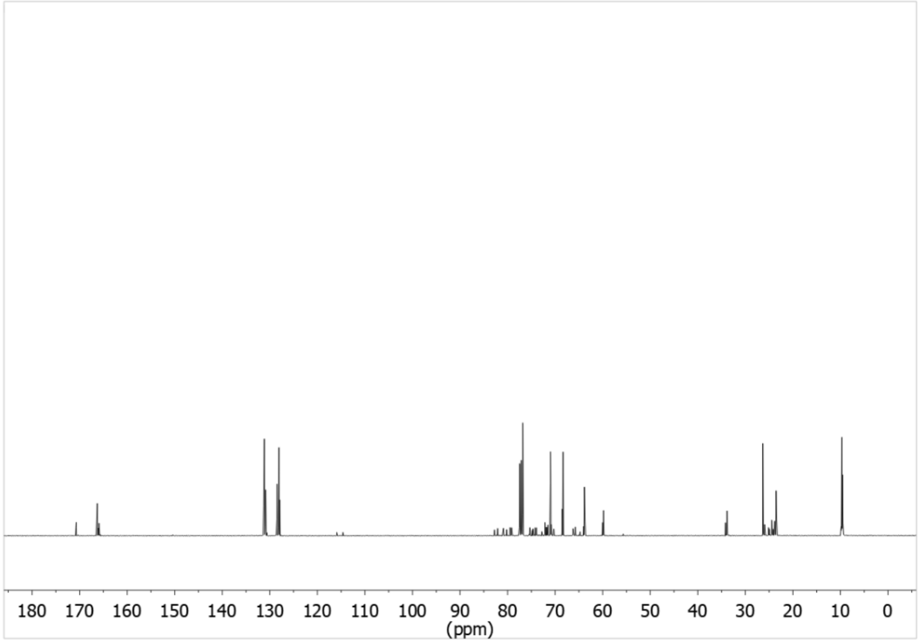

Figure S23. ^{13}C NMR spectrum (101 MHz, $CDCl_3$) of hydroxybutyl acrylate ([4C-1]) and 1-hydroxybutan-2-yl acrylate ([4C-2]). Carbonyl carbons of the acrylate esters appear at δ 170.78, 170.69, 166.39, and 166.28 ppm. Olefinic carbons are located at δ 131.01 and 128.28 ppm. Signals between δ 76.81 and 63.84 ppm correspond to oxygenated methylene and methine carbons, helping to differentiate the isomers. Aliphatic carbons appear between δ 26.33 and 7.43 ppm.

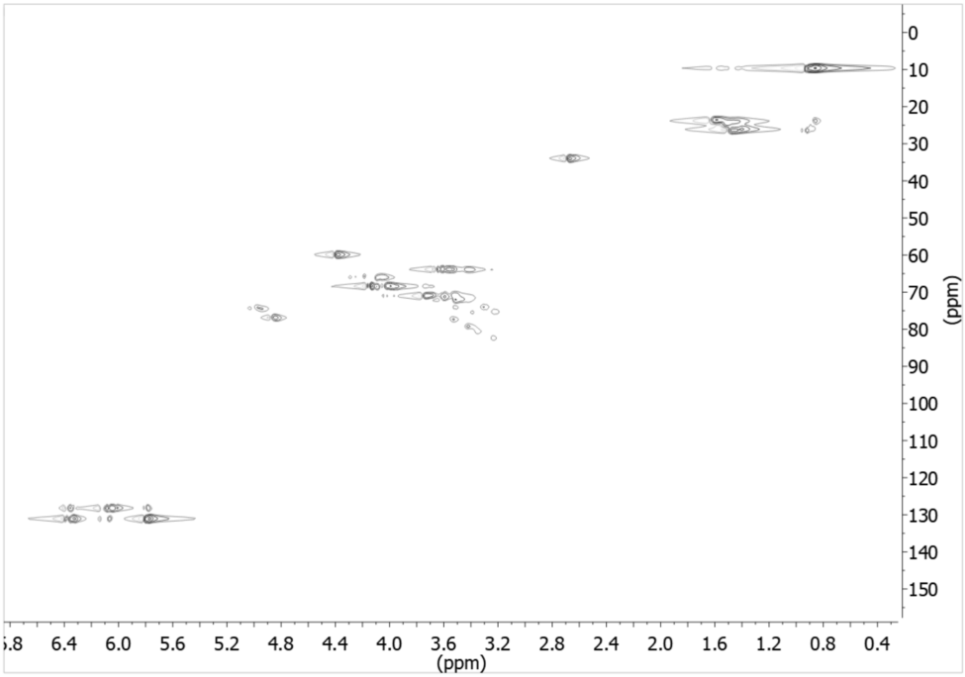

Figure S24. HSQC spectrum of hydroxybutyl acrylate ([4C-1]) and 1-hydroxybutan-2-yl acrylate ([4C-2]) in $CDCl_3$. Clear ^1H–^{13}C one-bond correlations enable assignment of acrylate vinyl protons and their corresponding carbons (~131 and ~128 ppm), as well as the hydroxylated CH_2 and CH groups (~76–64 ppm), distinguishing between linear and branched hydroxyl positions.

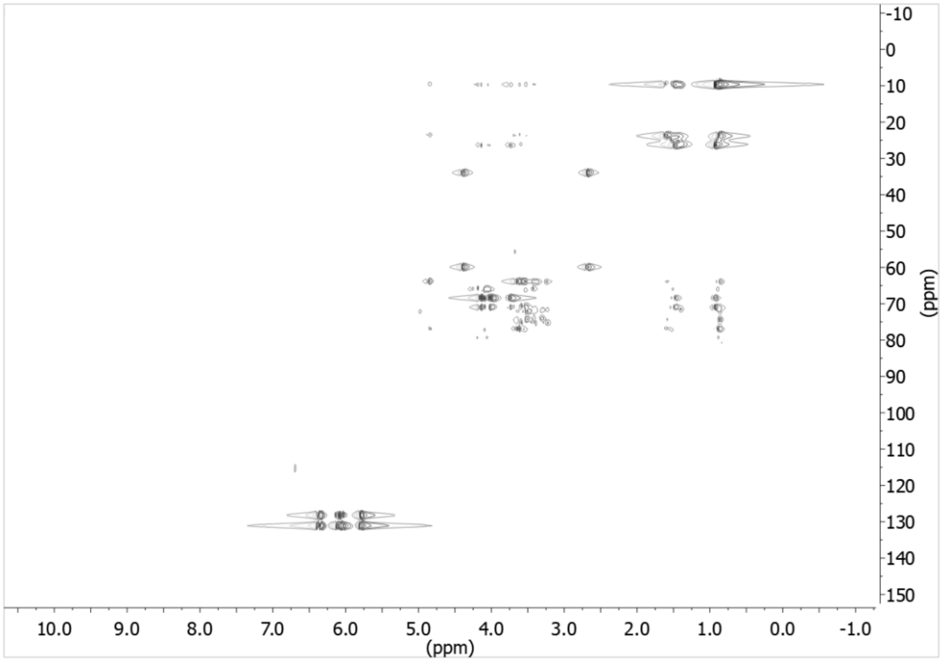

Figure S25. HMBC spectrum of hydroxybutyl acrylate ([4C-1]) and 1-hydroxybutan-2-yl acrylate ([4C-2]). Long-range ^{1}H–^{13}C correlations from the vinyl protons to the carbonyl carbons confirm acrylate structure. Cross-peaks between hydroxyl-bearing protons and adjacent carbons support differentiation of the substitution pattern.

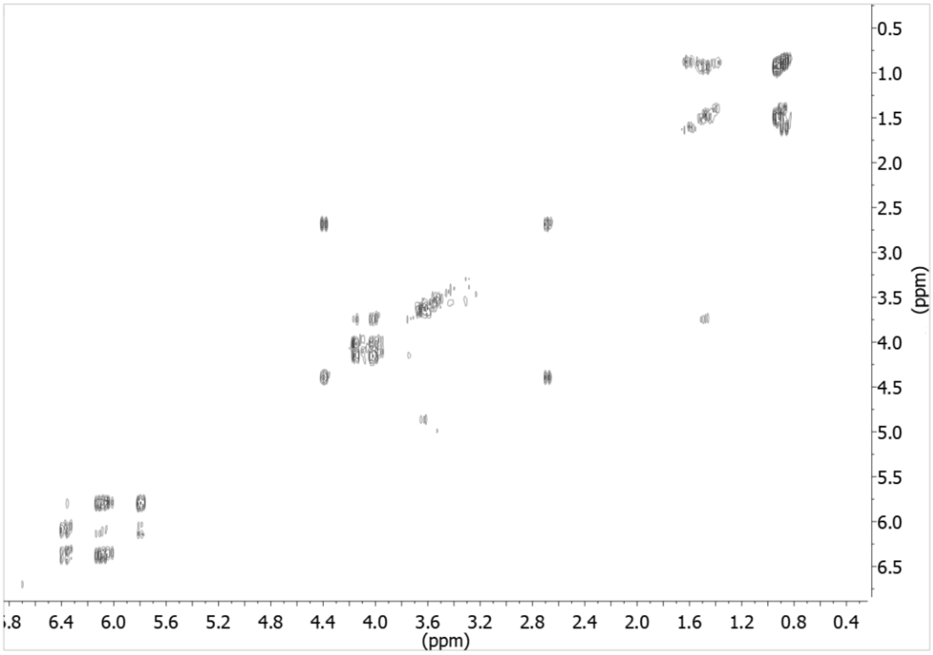

Figure S26. ^{1}H–^{1}H COSY spectrum of hydroxybutyl acrylate ([4C-1]) and 1-hydroxybutan-2-yl acrylate ([4C-2]). Coupling between vinyl protons, as well as correlations between hydroxylated and aliphatic methylenes. These patterns support the assignment of both regioisomers and establish proton connectivity along the chains.

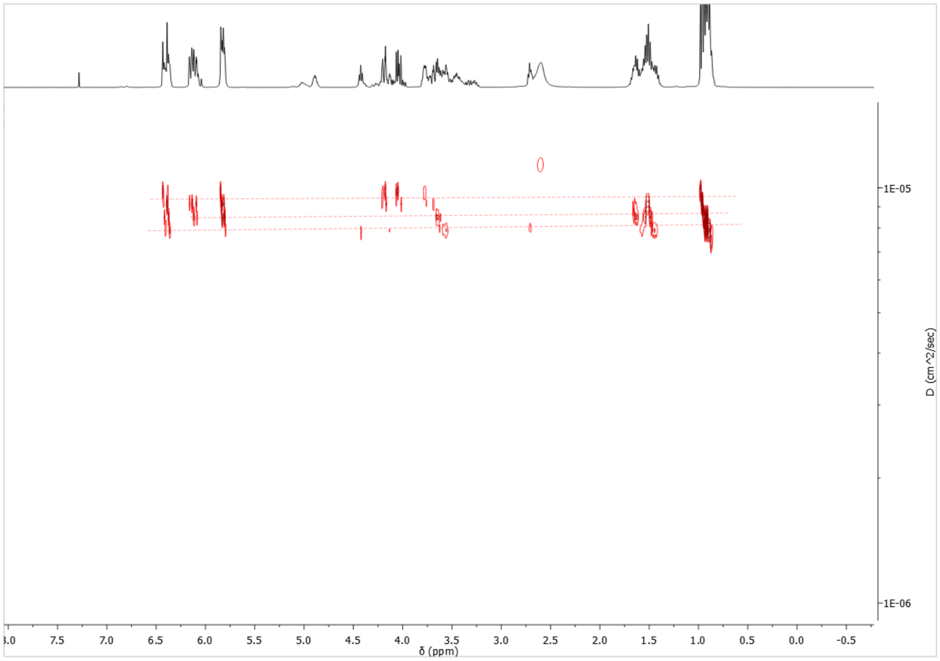

Figure S27. DOSY spectrum of hydroxybutyl acrylate ([4C-1]) and 1-hydroxybutan-2-yl acrylate ([4C-2]). Both products display single diffusion coefficients, indicating monomeric purity and the absence of significant polymerization or oligomer formation under the analytical conditions.

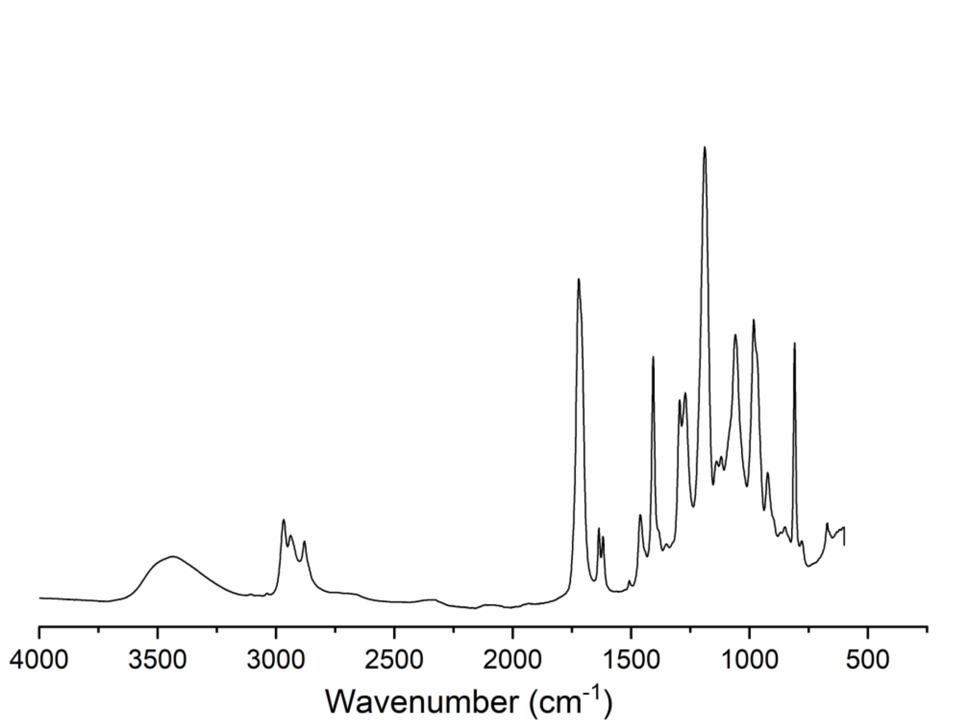

Figure S28. FTIR spectra of hydroxybutyl acrylate ([4C-1]) and 1-hydroxybutan-2-yl acrylate ([4C-2]). Characteristic bands include broad O–H stretching around 3400 cm^{-1}, C=O stretching at ~1720 cm^{-1}, and strong C–O stretching between 1150–1050 cm^{-1}, confirming the presence of hydroxyl and ester functionalities.

4. Polymerization

4.1. Size Exclusion Chromatography (SEC) Measurements

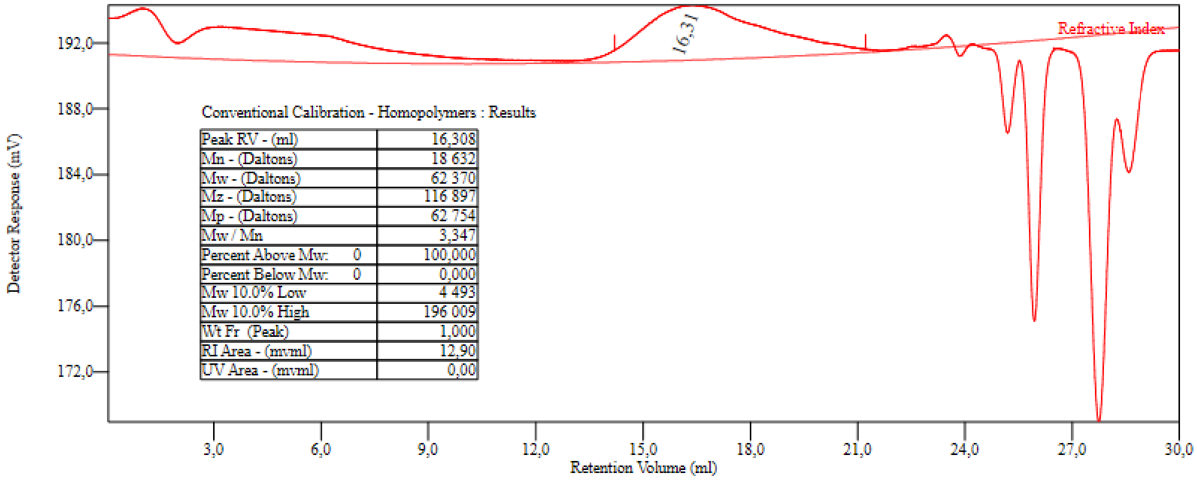

Figure S29. SEC elugram of poly([2C]) in $CHCl_3$. The trace displays a monomodal molecular weight distribution with an Mn of 18 600 and a dispersity of 3.3, indicating successful polymerization.

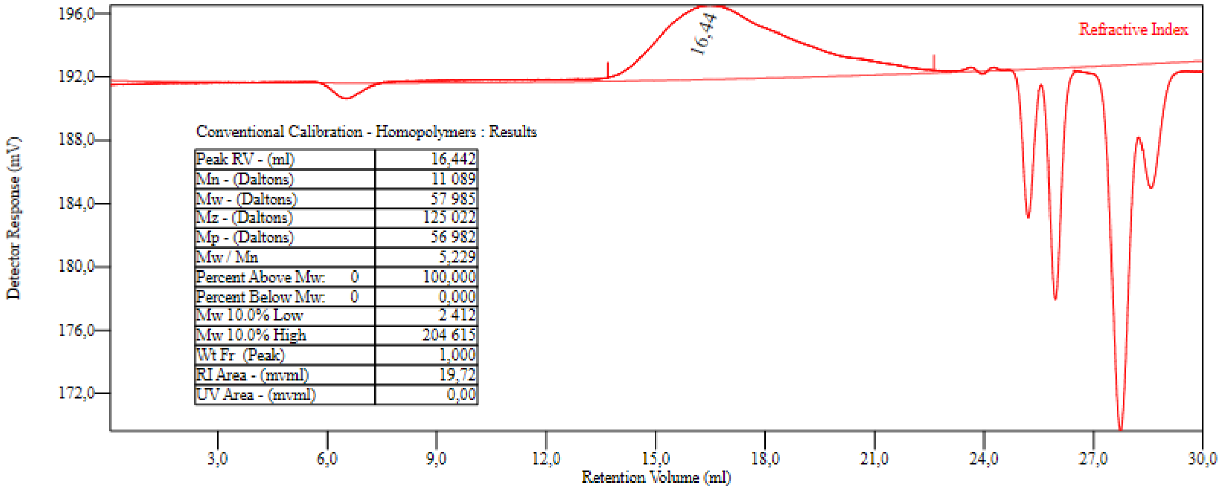

Figure S30. SEC elugram of poly([3C]) in $CHCl_3$. The polymer exhibits moderate Mn of 11 100 with a relatively broad distribution of 5.2.

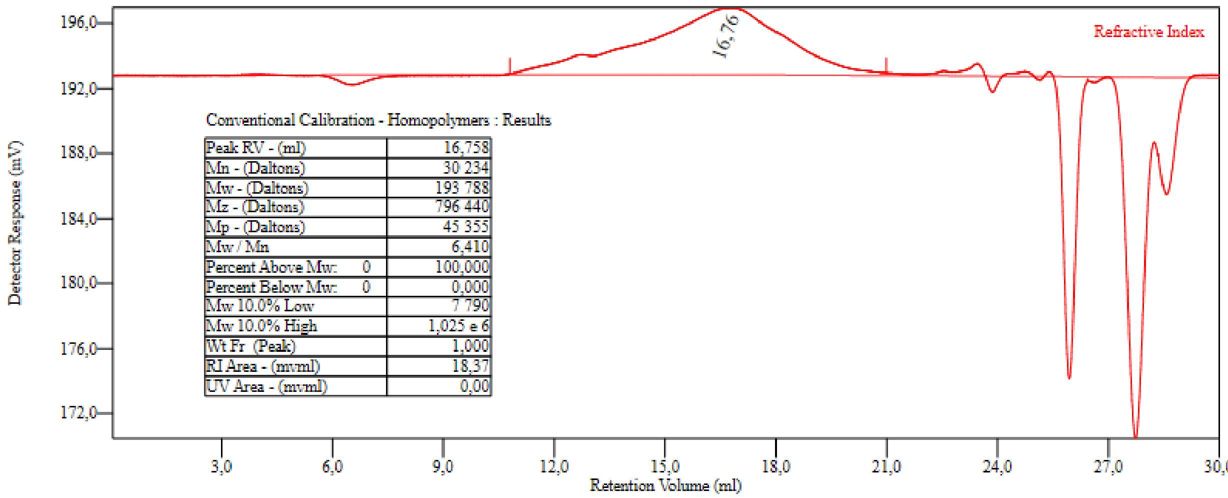

Figure S31. SEC elugram of poly([4C]) in $CHCl_3$. The polymer exhibits a Mn of 30 200 with a relatively broad distribution of 6.4.

4.2. Differential scanning calorimetry (DSC)

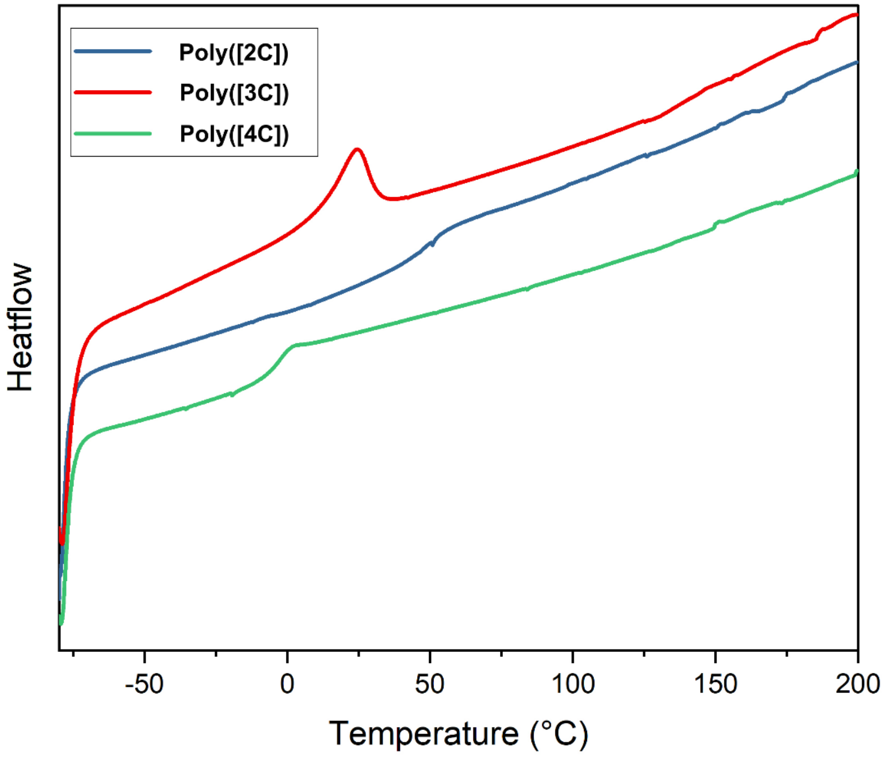

Figure S32. DSC thermograms of poly([2C]), poly([3C]), and poly([4C]). The polymers display distinct glass transition temperatures (Tg), with poly([2C]) showing the highest Tg due to its rigid cyclic structure, and poly([3C]) the lowest, consistent with its flexible decyl side chains.

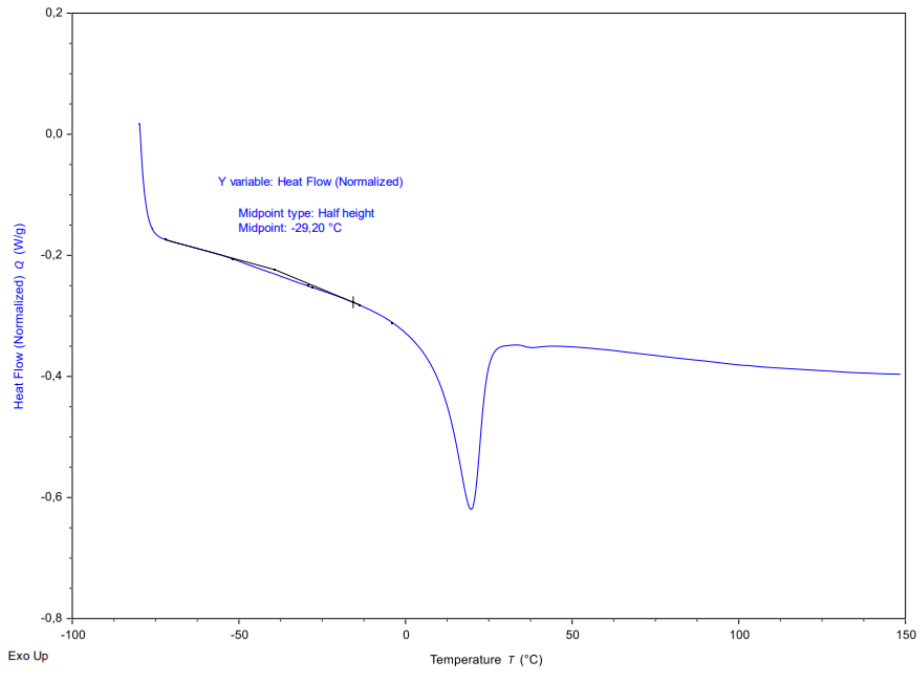

Figure S33. DSC trace of poly([3C]) using an increased heating rate and larger sample size. The glass transition temperature is observed at –29 °C, consistent with its long, linear side chains acting as internal plasticizers and reducing chain packing efficiency

4.3. Pure poly([2C])

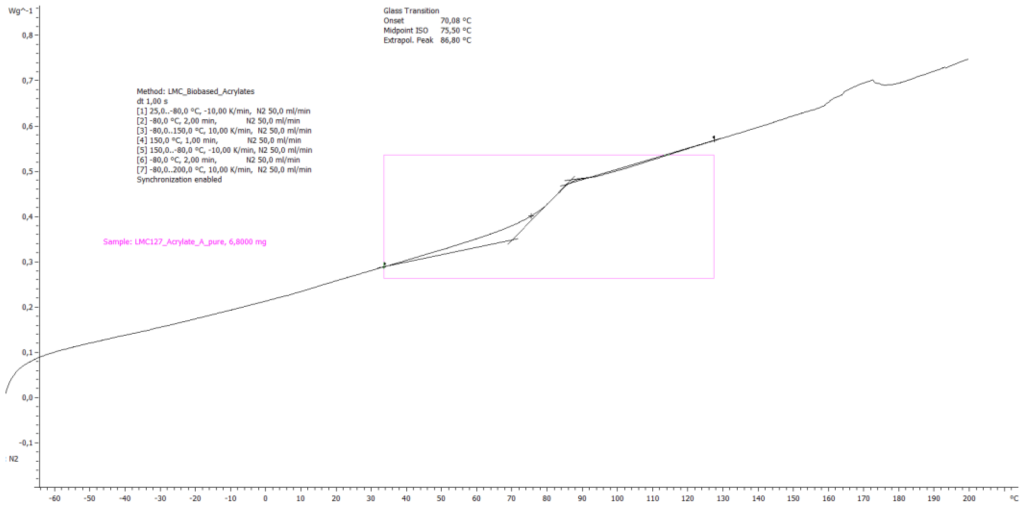

Figure S34. DSC analysis of poly([2C]) synthesized from a purified batch of [2C] monomer. A 20 °C increase in Tg compared to the crude monomer batch highlights the plasticizing effect of residual oligomers and impurities on the polymer's thermal properties.

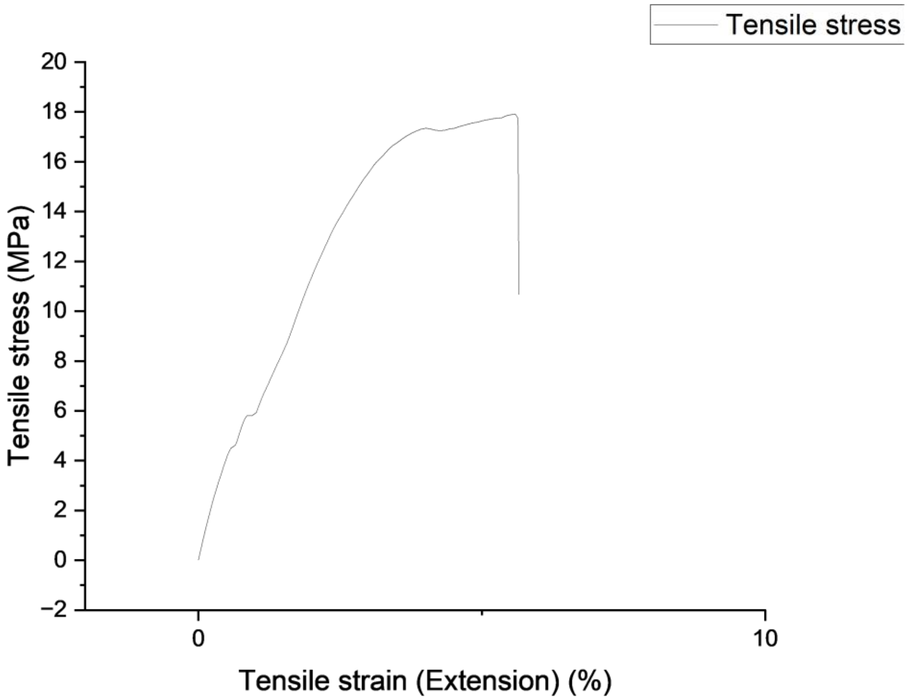

Figure S35. Tensile test of purified poly([2C]) polymer. The material exhibits a tensile strength of 18 MPa, a strain at break of 5.6%, and a Young's modulus of 630 MPa. These mechanical properties are consistent with samples prepared from crude [2C], confirming that monomer purity primarily influences Tg but not tensile behavior.

5. Purified Cyclohexene-oxide acrylate and RAFT Polymerization

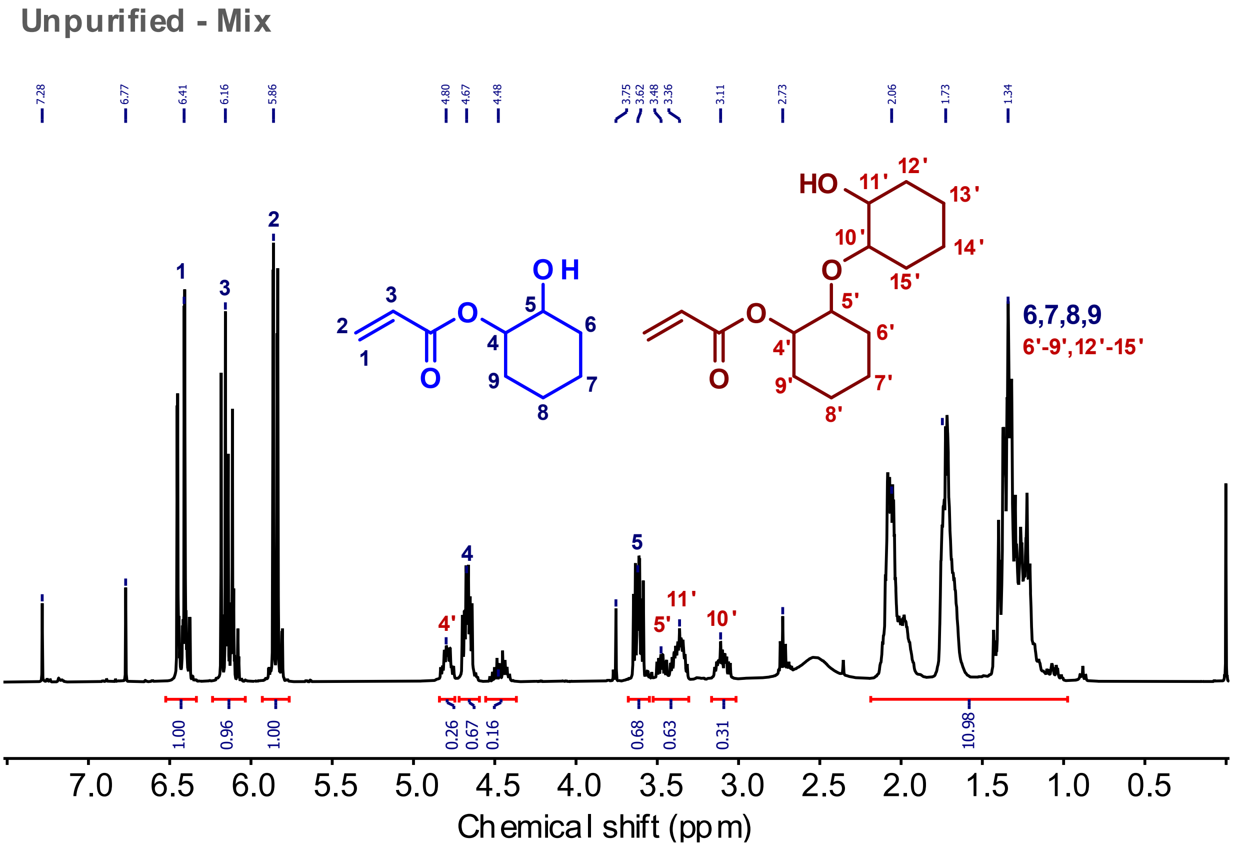

Figure S36. ^{1}H NMR of the initial product from the ring-opening acrylation of cyclohexene oxide acrylate. The spectra show the formation of two main products, namely 2-hydroxycyclohexyl acrylate and 2-((2-hydroxycyclohexyl)oxy)cyclohexyl acrylate.

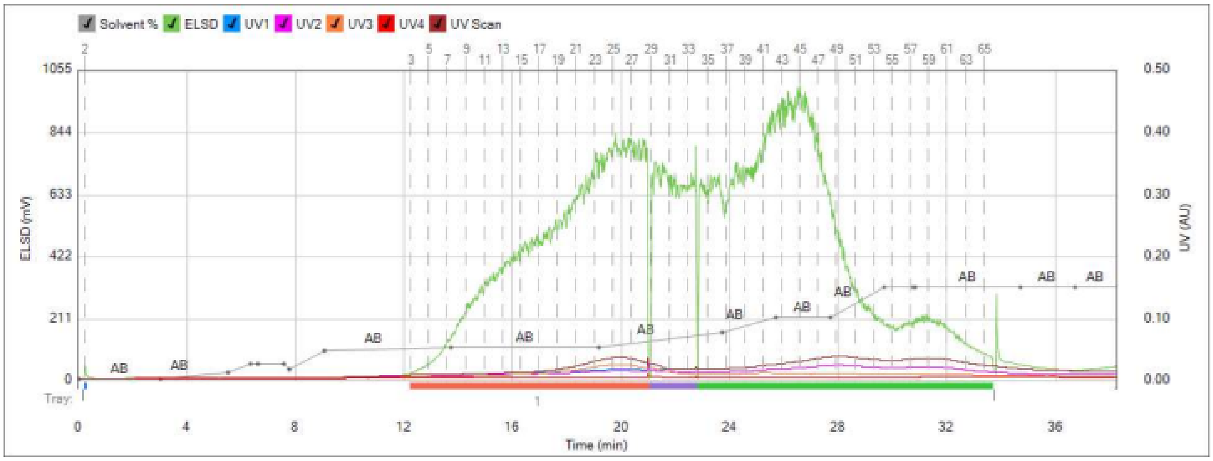

1 - 3D6B

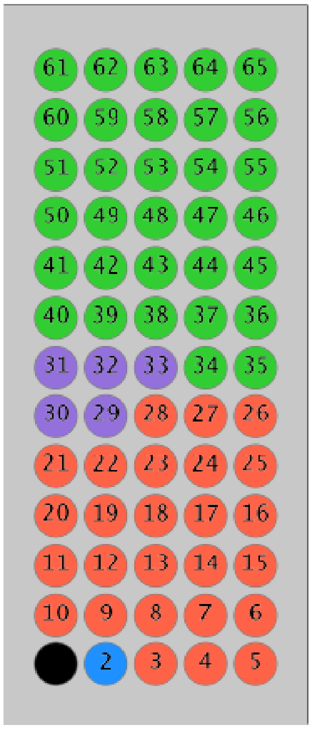

Gradient Table

	Min	Solvents	% 2nd
1	0.0	AB	0
2	3.0	AB	0
3	2.5	AB	2
4	0.8	AB	5
5	0.2	AB	5
6	1.0	AB	5
7	0.2	AB	3
8	0.0	AB	3
9	1.3	AB	9
10	4.7	AB	10
11	5.5	AB	10
12	4.6	AB	15
13	2.0	AB	20
14	2.0	AB	20
15	2.0	AB	30
16	1.1	AB	30
17	0.1	AB	30
18	3.9	AB	30
19	2.0	AB	30
20	5.0	AB	30

Vial Mapping Table

Peak #	Start Tray:Vial	End Tray:Vial
1	1:2	1:2
2	1:3	1:28

Figure S37. Using automatic collum enabled us to separate the monomer and the dimer, n-heptane EtOAc mixture.

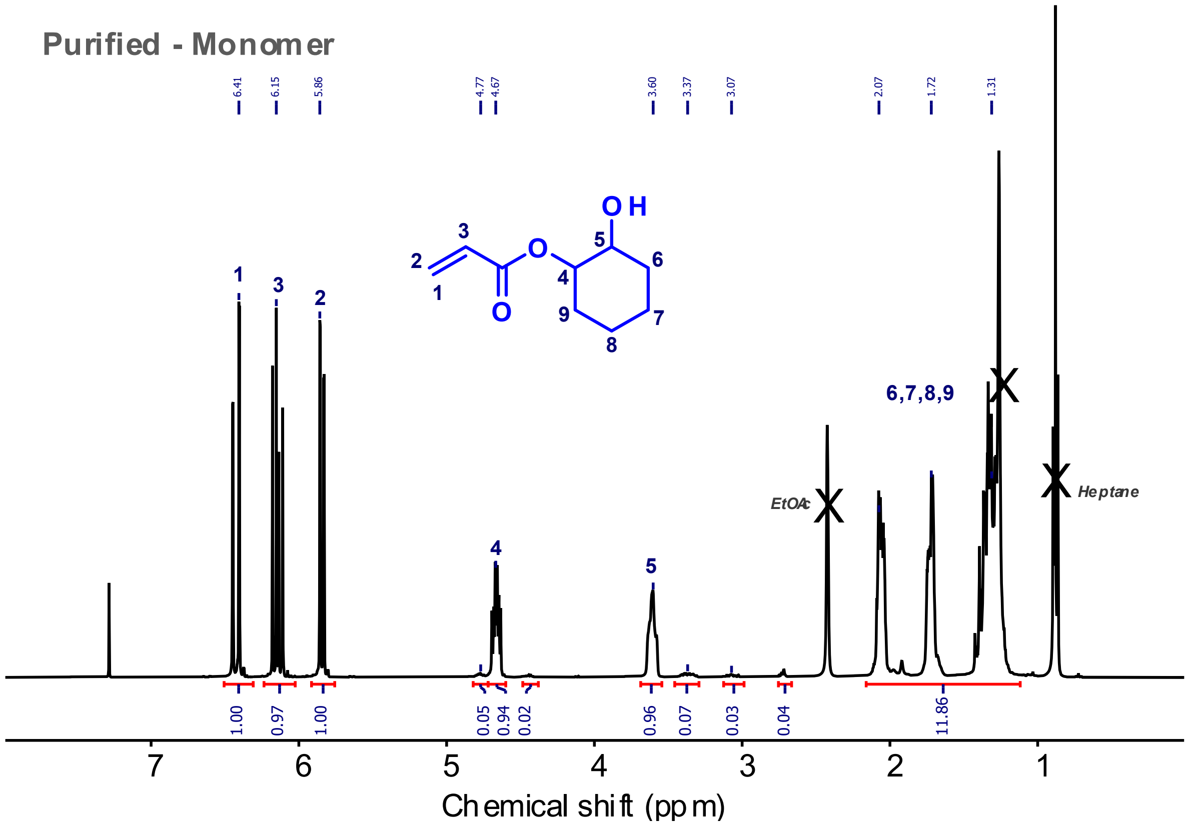

Figure S38. ^{1}H NMR of the purified monomer, showing mainly isolation of the monomer product, 2-hydroxycyclohexyl acrylate

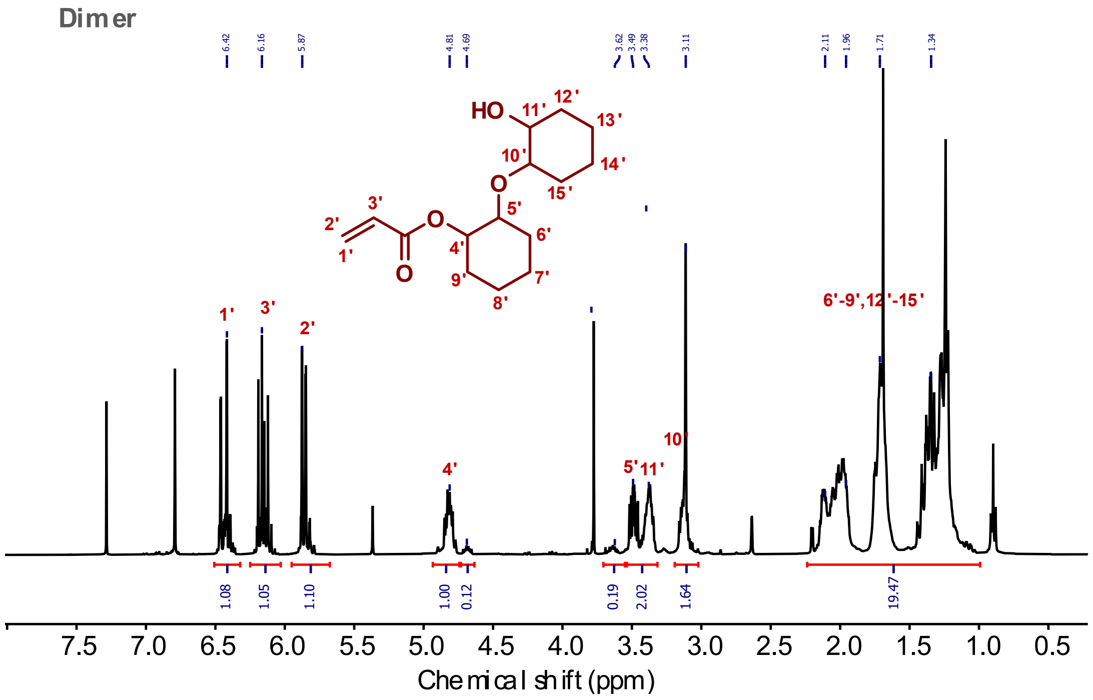

Figure S39. ^{1}H NMR of the purified monomer, showing main isolation of the dimer monomer product, 2-((2-hydroxycyclohexyl)oxy)cyclohexyl acrylate.

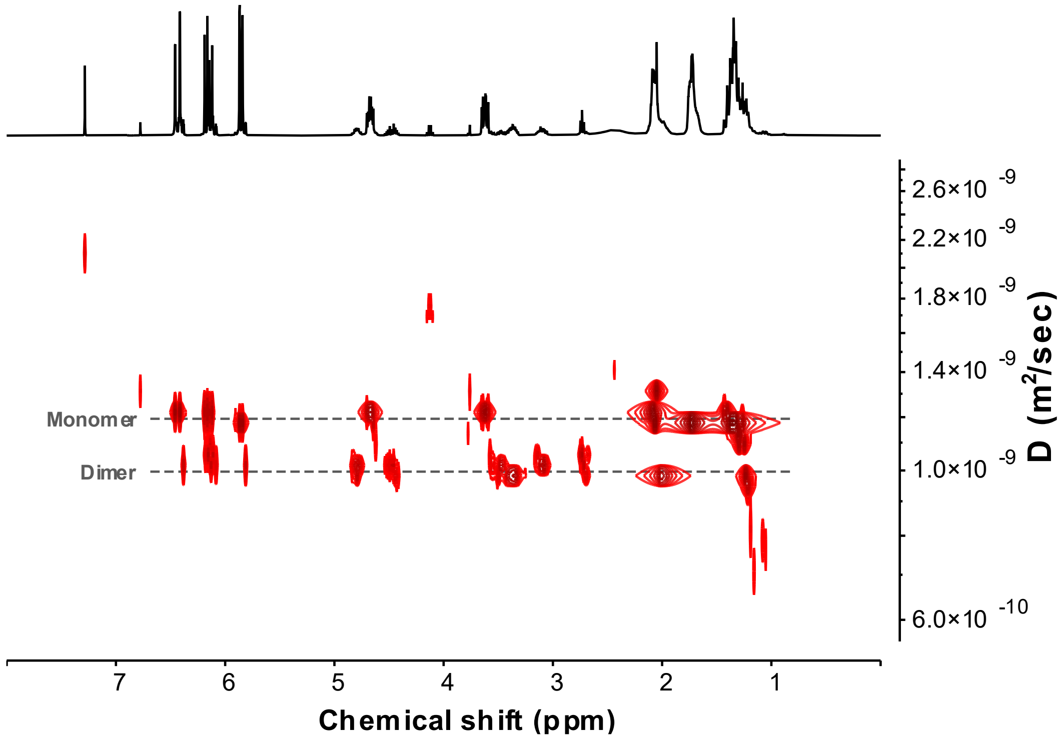

Figure S40. DOSY NMR of the initial product from the ring-opening acrylation of cyclohexene oxide acrylate. The spectra show the formation of two main products, namely 2-hydroxycyclohexyl acrylate and 2-((2-hydroxycyclohexyl)oxy)cyclohexyl acrylate.

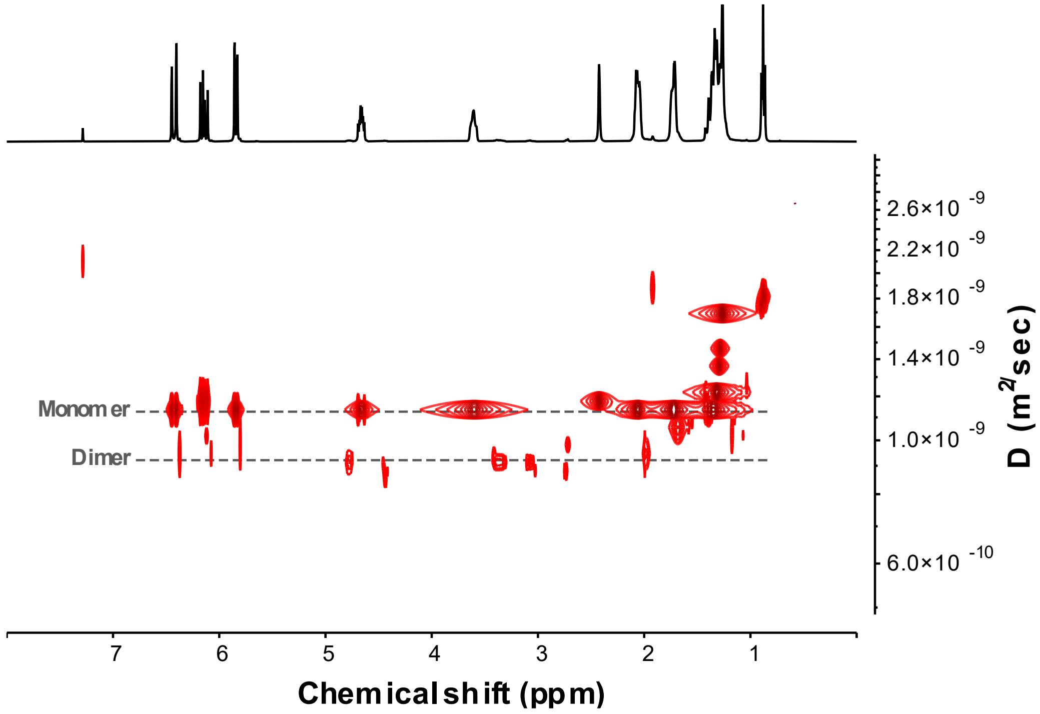

Figure S41. DOSY NMR of the purified monomer, showing mainly isolation of the monomer product, 2-hydroxycyclohexyl acrylate

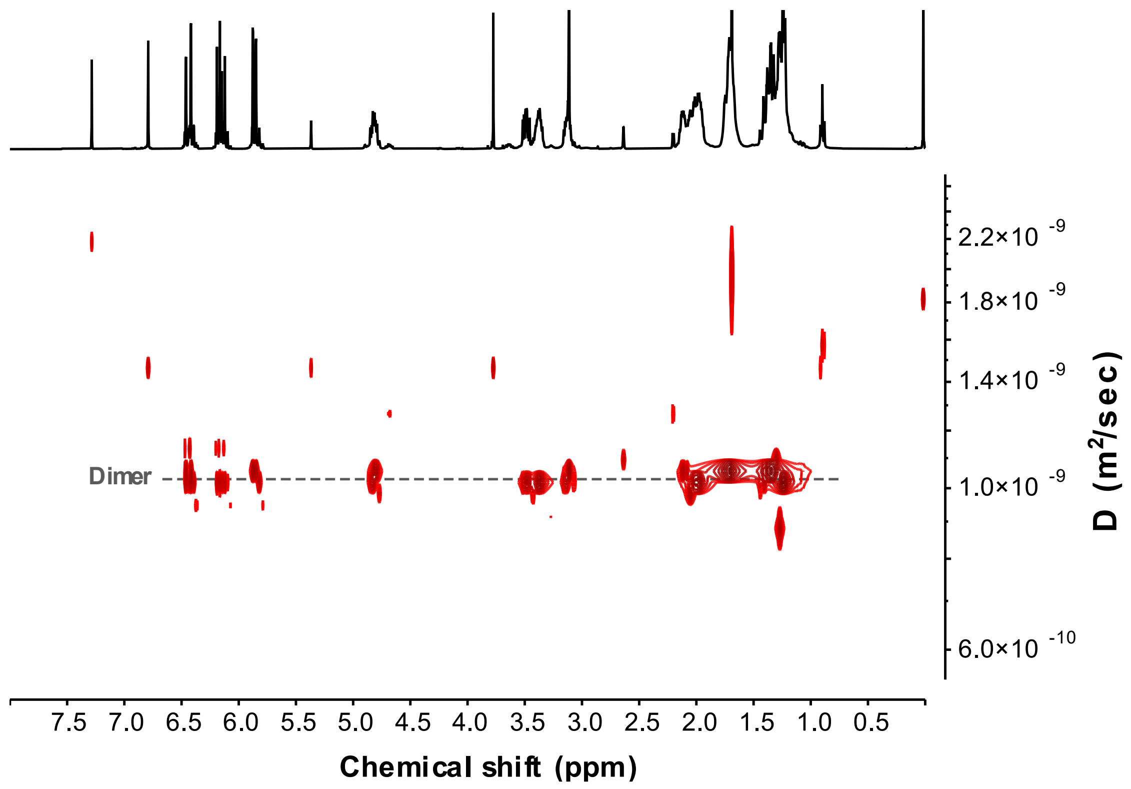

Figure S42. DOSY NMR of the purified monomer, showing main isolation of the dimer monomer product, 2-((2-hydroxycyclohexyl)oxy)cyclohexyl acrylate.

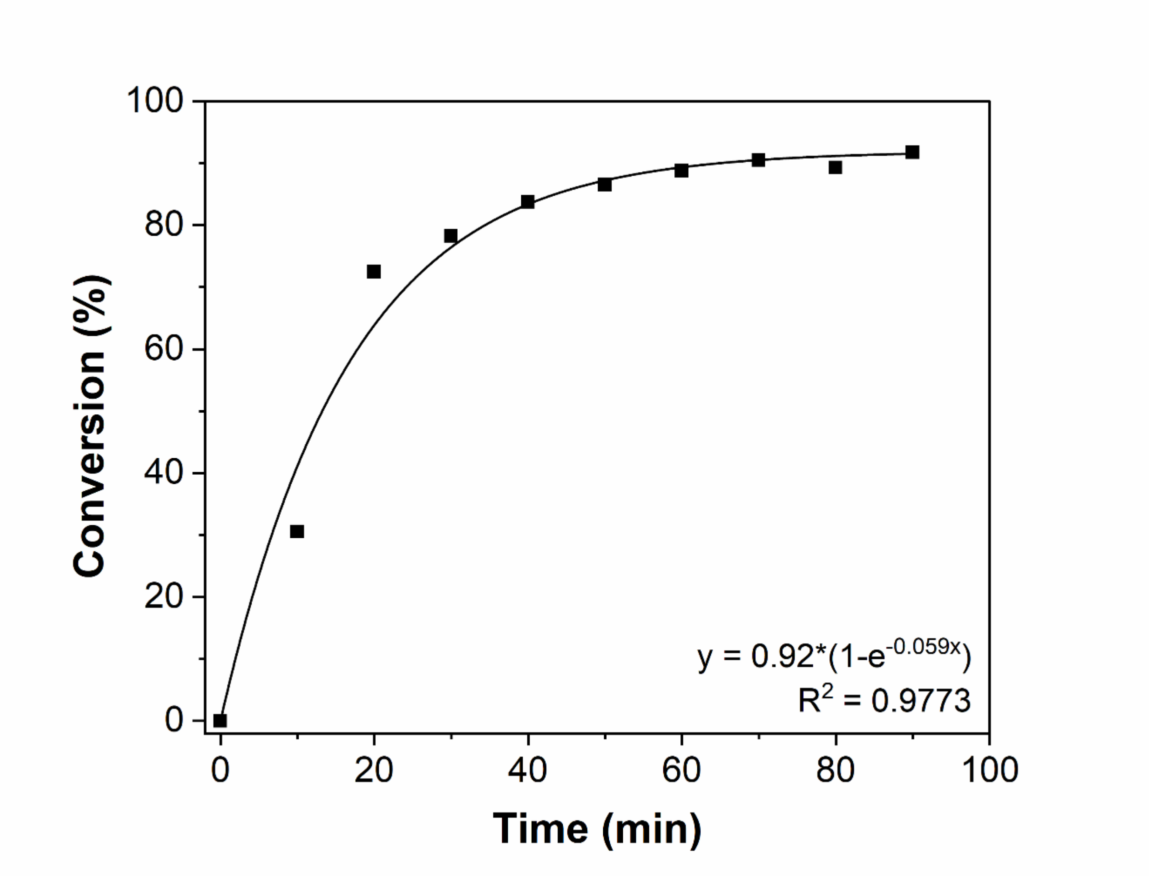

Figure S43. Conversion over time for RAFT polymerization of 2-hydroxycyclohexyl acrylate. Conversion was determined by ^1H NMR using DMF as an internal standard.

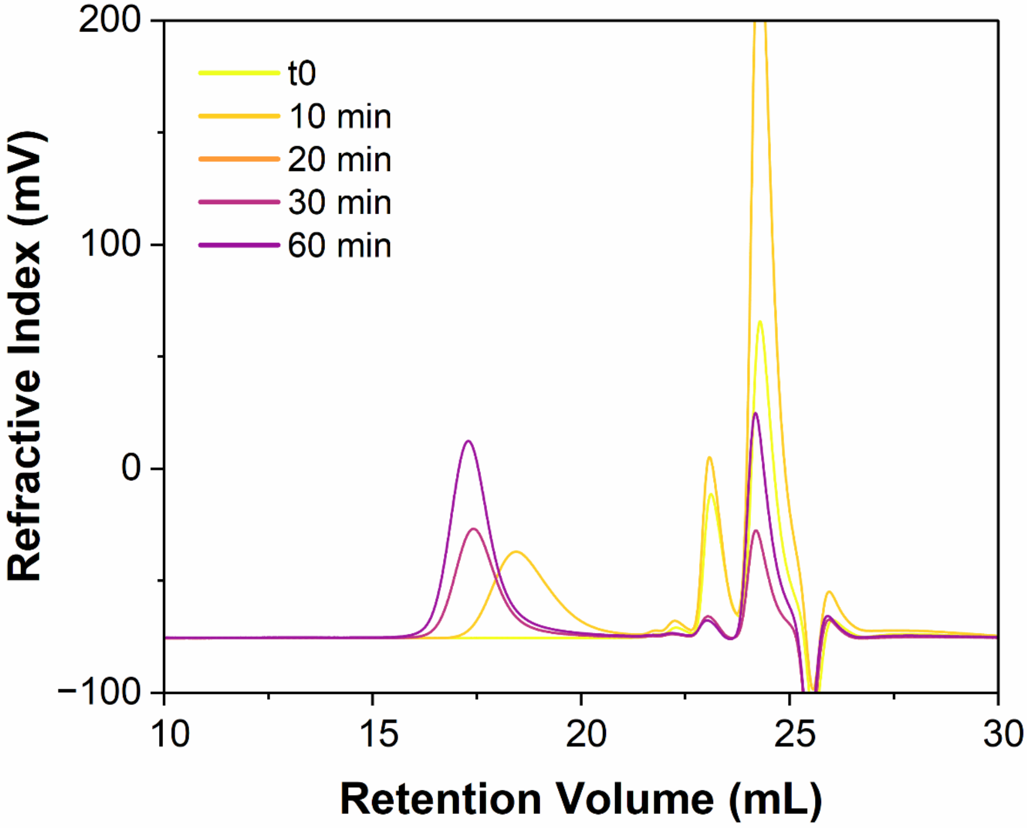

Figure S44. SEC traces for RAFT polymerization of 2-hydroxycyclohexyl acrylate. SEC was performed in CHCl3 using polystyrene standards.

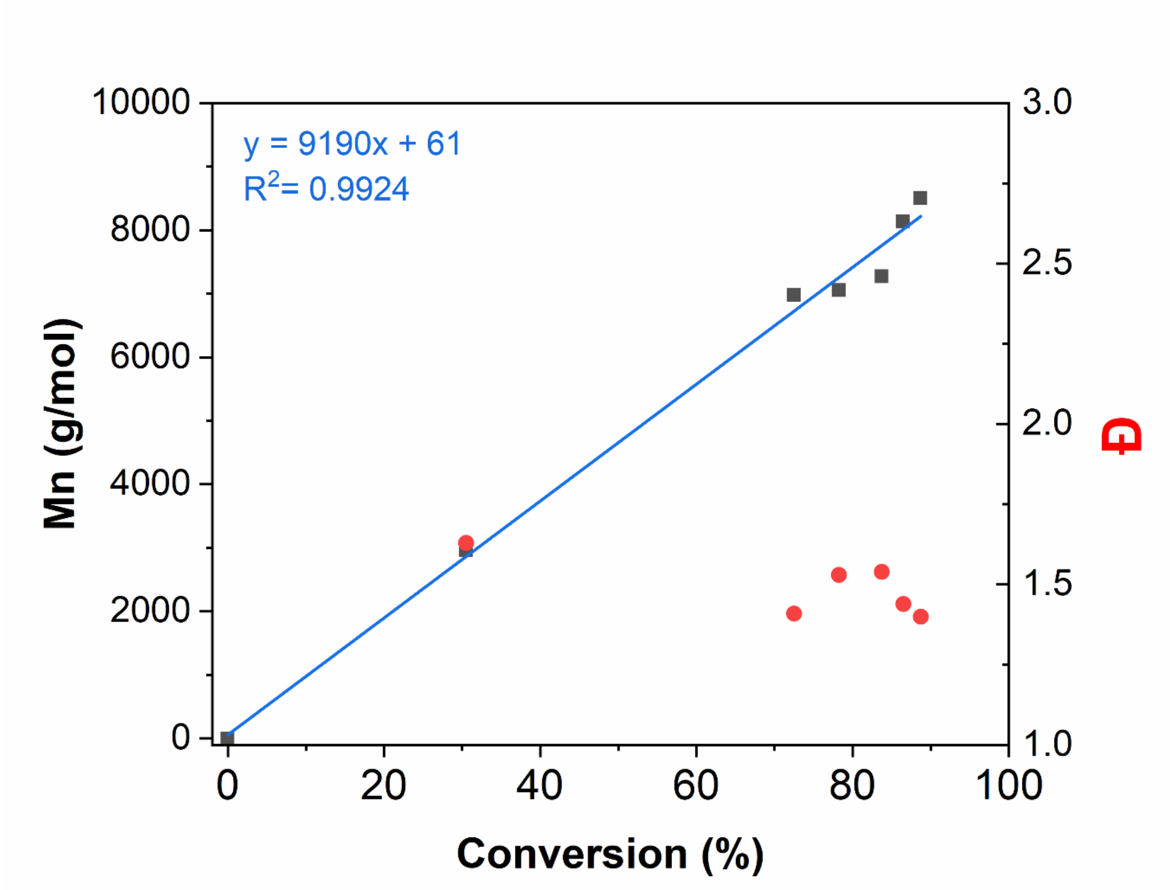

Figure S45. Mn and Đ against conversion
